# Chemostructurally Stable Polyionomer Coatings Regulate Proton-Intermediate Landscape in Acidic CO_2_ Electrolysis

**DOI:** 10.1021/jacs.5c01314

**Published:** 2025-07-29

**Authors:** Bárbara Polesso, Adrián Pinilla-Sánchez, Eman H. Ahmed, Anku Guha, Marinos Dimitropoulos, Blanca Belsa, Viktoria Golovanova, Lu Xia, Ranit Ram, Sunil Kadam, Aparna M. Das, Junmei Chen, Johann Osmond, Adam Radek Martínez, Melanie Micali, Esther Alarcón Lladó, F. Pelayo García de Arquer

**Affiliations:** † 172281 ICFO-Institut de Ciències Fotòniques, The Barcelona Institute of Science and Technology, Castelldefels (Barcelona) 08860, Spain; ‡ NRC-National Research Centre, Polymers and Pigments Department, Chemical industries research institute, Advanced Materials and Nanotechnology group, Cairo 12622, Egypt; § Center for Nanophotonics, NWO-Institute AMOLF, Science Park 104, 1098 XG Amsterdam, The Netherlands; ∥ Van’t Hoff Institute for Molecular Sciences (HIMS), University of Amsterdam, 1090 GD, Amsterdam, The Netherlands

## Abstract

CO_2_ electroreduction (CO_2_R) in acidic media offers a path to high carbon utilization via local carbonate regeneration. However, this proton-rich environment challenges achieving a combined selectivity and rate toward multicarbon (C_2+_) products due to proton and intermediate competition. Here, we demonstrate a strategy to modulate local protons and intermediates, at these settings, using a polyionomer coating over benchmark copper gas diffusion electrodes. The polyionomer integrates amine (−NH_*x*_) function from branched polyethylenimine (PEI) with sulfonate (−SO_3_^–^) and amphiphilic functions from PFSA. We show that their chemical structure enables H-bonding interaction, leading to a stereochemical assembly that retains a structure–property relationship through a wide pH range (2–14). PFSA domains modulate *CO intermediates and local [CO_2_]/[H_2_O] and K^+^ environment, while partially protonated amines provide further control over proton availability and intermediate stabilization, which in combination enhance C–C coupling. When implemented in a flow cell (0.5 M K_2_/H_2_SO_4_, pH = 2), the optimized polyionomer coating enables a C_2+_ Faradaic efficiency of 61% at a single-pass CO_2_ utilization of 84%, including a conversion efficiency of 64% toward C_2+_, at a current density of at 0.3 A cm^–2^—an improvement of almost 30% in C_2+_ selectivity and 35% in carbon utilization compared to monofunctional coatings. These findings expand the toolbox of strategies to modulate CO_2_R microenvironments toward improved performance.

## Graphical abstract

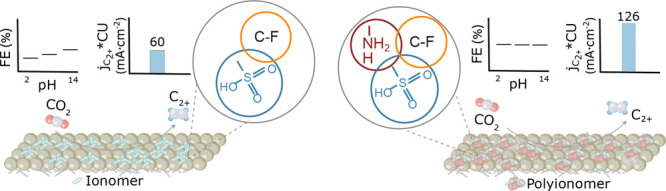

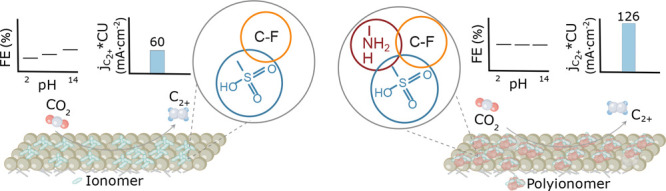

## Introduction

Electrochemical CO_2_ reduction (CO_2_R) offers a path to mitigate global greenhouse emissions by converting atmospheric and waste CO_2_ into widely used chemicals such as syngas, formic acid, methane, ethanol, and ethylene, among others, using renewable and low carbon electricity.^1–5^

Among these molecules, ethylene and ethanol (multicarbon, C_2_ products) are attractive in view of their industrial relevance and potential carbon footprint reduction.^6^ Ethylene is widely used as a precursor in the polymer industry and stands out as the largest market size and most valuable product, contributing as the primer product to CO_2_ emissions. Ethanol has a high volumetric energy density and can be incorporated into existing fuel supply chains.^1,4–7^ Further progress in the performance toward these are still necessary to approach technoeconomic viability.^4–6,8^

Best performance toward C_2_ products has been consistently achieved using copper gas diffusion electrodes. Initial progress in alkaline catholytes^3,9–12^ was shown to be impractical due to the spontaneous consumption of CO_2_ by OH^–^ into (bi)­carbonates, limiting carbon conversion efficiencies (<5%) and overall process viability.^13–15^

This prompted the search for alternatives such as operation in neutral membrane electrode assemblies (MEA), tandem reaction schemes, and carbonate regeneration via local proton replenishment.^16–19^ The latter can be implemented using reversely biased bipolar membranes^20^ or, directly, by operating CO_2_R in acid media.^13,14,21–27^ In such a configuration, (bi)­carbonates would be converted back to CO_2_ by reacting with protons replenished from the anode.^14,25^ This, however, favors the competing hydrogen evolution reaction (HER), limiting CO_2_R rates (Figure 1a).^13,14,21–26^

**Figure 1:**
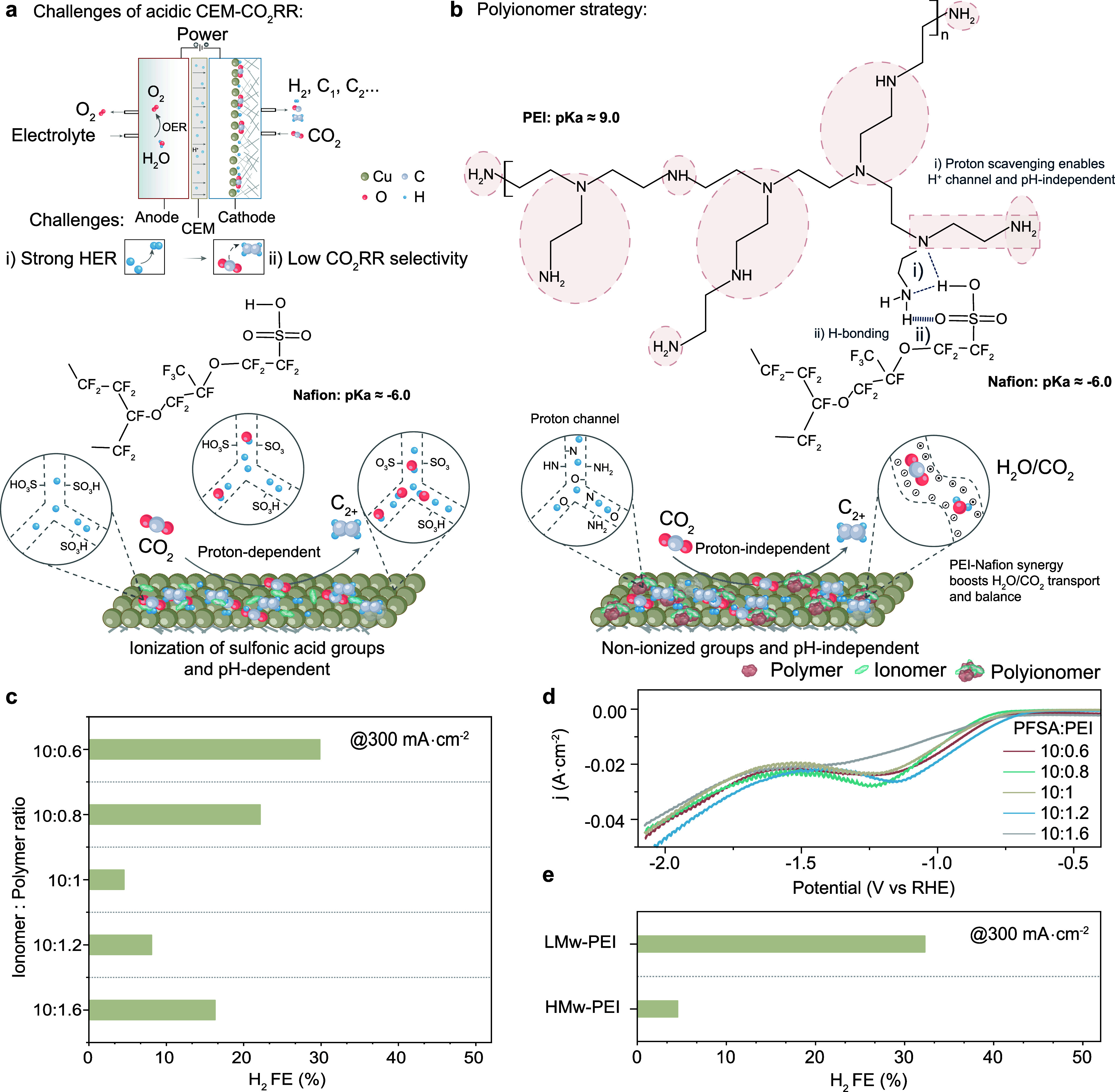
Polyionomer coatings retain chemostructural function in acid to jointly address local protons and CO_2_R intermediates. (a) CO_2_R challenges and mechanism in acidic media. (b) Chemical structure, orientation, and p*K*_a_ values contribute to maintaining the optimal structure–property function of the polyionomer across a wide range of pH values. This is attributed to the presence of proton channels, hydrogen bonding, and [CO_2_]/[H_2_O] balance which are absent in Cu/ionomer coatings. The highlighted regions in PEI indicate interactions between branched PEI (9) and PFSA (1). (c) Hydrogen Faradaic efficiency (FE) of different ionomer:polymer ratios. (d) Linear sweep voltammetry (LSV) curves indicating proton barrier effect of different ratios of branched PEI with Ar (40 mL min^–1^) and scan rate of 50 mV s^–1^. (e) Hydrogen Faradaic efficiency (FE) of Cu/polyionomer with low (LMw-PEI) and high (HMw-PEI) molecular weights of PEI.

Initial progress to overcome this competition largely relied on local pH control. This happens naturally at increasing current densities: as protons get consumed, and their transport is increasingly limited, the proton source shifts from hydronium to water, leading to higher local pH. This may enable CO_2_R depending on the reaction environment.^14,27–29^ In neutral and alkaline electrolytes, the local microenvironment can be tuned in the electrode–electrolyte interface using different organic coatings, manipulating CO_2_R intermediates and product distribution.^25,26,30–34^ For example, amine-functionalized polymers may act as local proton scavengers and CO_2_ concentrators, boosting C_2+_ product yields on copper gas diffusion electrodes.^35–37^

However, we posited that surface modification strategies developed for alkaline and neutral CO_2_R may not be directly applicable to acidic electrolytes. In neutral and alkaline media, polymer/polyionomer modifiers have been primarily optimized to control OH^–^ diffusion, enhance CO_2_ concentration, and tune hydrophobicity.^38,39^ Crucially, these designs do not directly address local proton activity, which under acidic conditions becomes the dominant factor driving competitive HER. Without a mechanism to buffer or regulate proton access at the catalyst interface, conventional coatings lose efficacy and selectivity in acid-fed CO_2_R.

To overcome these challenges, recent approaches such as cation-group augmentation and organic thin films have separately demonstrated control over proton-flux, mitigation or intermediate stabilization in strongly acidic CO_2_R.^27,29,31,40^

We hypothesized that the high proton concentration and distinct ionic environment in acid may not only alter reaction pathways—favoring hydrogen evolution over CO_2_R—but also disrupt structure–property–function relationships of polymer coatings. Our preliminary results revealed varying hydrophobicity of PFSA coatings as a function of pH (Figure S1), which could be compatible with this picture.

To bridge this gap, we sought to design a chemostructurally stable coating capable of “locking in” multifunctionality across the wide pH swings characteristic of acid-fed CO_2_R. To this end, we turned our attention to PFSA ionomers (e.g., Nafion) and branched polyethylenimine (PEI). The former, in view of their ability to improve CO_2_R by virtue of cation concentration and water management; the latter, because of their wide primary, secondary, and tertiary amine functions.

In acidic conditions, the protonation ratio of amine groups is higher compared to that in neutral and alkaline pHs.^41^ We hypothesized that this could enable function beyond conventional CO_*x*_ stabilization, as the interaction of amines with −SO_3_^–^ groups could be harnessed to control ionomer assembly and structure–property function in acid.

Owing to the chemical structure, orientation, and p*K*_a_ values, the amine groups in PEI (p*K*_a_ ≈ 9.0)^42^ and −SO_3_H group of PFSA (p*K*_a_ ≈ −6)^43^ via proton scavenging form a stable and favorable 5-membered ring structure along with H-bonding in specific branched positions (Figure 1b). Thus, the polyionomer structure stabilizes in a typical 1:9 ratio of PEI and PFSA. Unlike for monofunctional coatings, which show a highly pH-sensitive CO_2_R product distribution, polyionomers retain HER suppression within pH 2–14 (Figure S2). The optimized polyionomer coatings (PEI and PFSA in 1:10) implemented over benchmark poly­(tetrafluoroethylene) (PTFE)/polycrystalline copper gas diffusion electrodes further enable the simultaneous control over *CO intermediates and reaction modulators ([CO_2_]/[H_2_O] and CO_2_ potassium activation), supported by in situ Raman spectroscopy.

When implemented in a flow cell (0.5 M K_2_/H_2_SO_4_, pH = 2), they achieve a single pass CO_2_ conversion of 84% at industrially relevant current densities (0.3 A cm^–2^) and selective C_2+_ production of 61%. This represents an improvement of almost 30% in C_2+_ selectivity and 35% in carbon utilization compared with conventional monofunctional coatings.

## Results

We began by assessing the effect of PEI (polymer) incorporation on H_2_ suppression for a fixed amount of PFSA (ionomer) and loading. Scanning electron micrographs (SEM-EDX) and atomic force microscopy (AFM) revealed a homogeneous coating of PSFA-PEI binders over the entire catalyst surface (Figures S3–S5). An ionomer:polymer ratio of 10:1 results in a significant suppression of H_2_ generation at 0.3 A cm^–2^ (Figure 1c and Figure S6). Polarization curves (Figure 1d) indicate a proton barrier effect depending on ionomer:polymer ratio, revealing a depletion of H_3_O^+^ at lower currents for 10:1 compared to others.^29^

To address how the amount of amine groups could interfere in CO_2_ activation and hydrogen bonding close to the surface,^30^ we further compared the extent of HER suppression for branched high and low molecular weight (Mw) polyethylenimine (PEI). HER was more suppressed with high Mw PEI (HMw-PEI) (Figure 1e and Figure S7), which could be correlated with a thicker proton-resistant layer due to a larger number of repeating units.^44^

To assess the individual role of the polymer and ionomer on microenvironment modulation, we prepared three different electrode configurations: two in a bilayer configuration (copper/polymer/ionomer and copper/ionomer/polymer) and one copper/polyionomer (Figure S8).

Comparing again the H_2_ selectivity at 0.3 A cm^–2^ (Figure S9), the polyionomer shows the highest H_2_ suppression (Figure S9). Both bilayer configurations also suggest the presence of regions with high local acid penetration, allowing the HER to dominate the overall reaction, probably blocking the diffusion of CO_2_ to desired products. This is supported by the plateau of HER current in the bilayer configuration (Figure S10).

To get more insights on the chemical configuration of the polyionomer, the interaction between their functional groups, and structure–property function, we performed a combination of X-ray photoelectron spectroscopy (XPS), attenuated total reflectance-Fourier transform infrared (ATR-FTIR), contact angle, and atomic force microscopy (AFM)/Kelvin probe force microscopy (KPFM) (Figure 2) measurements.

**Figure 2:**
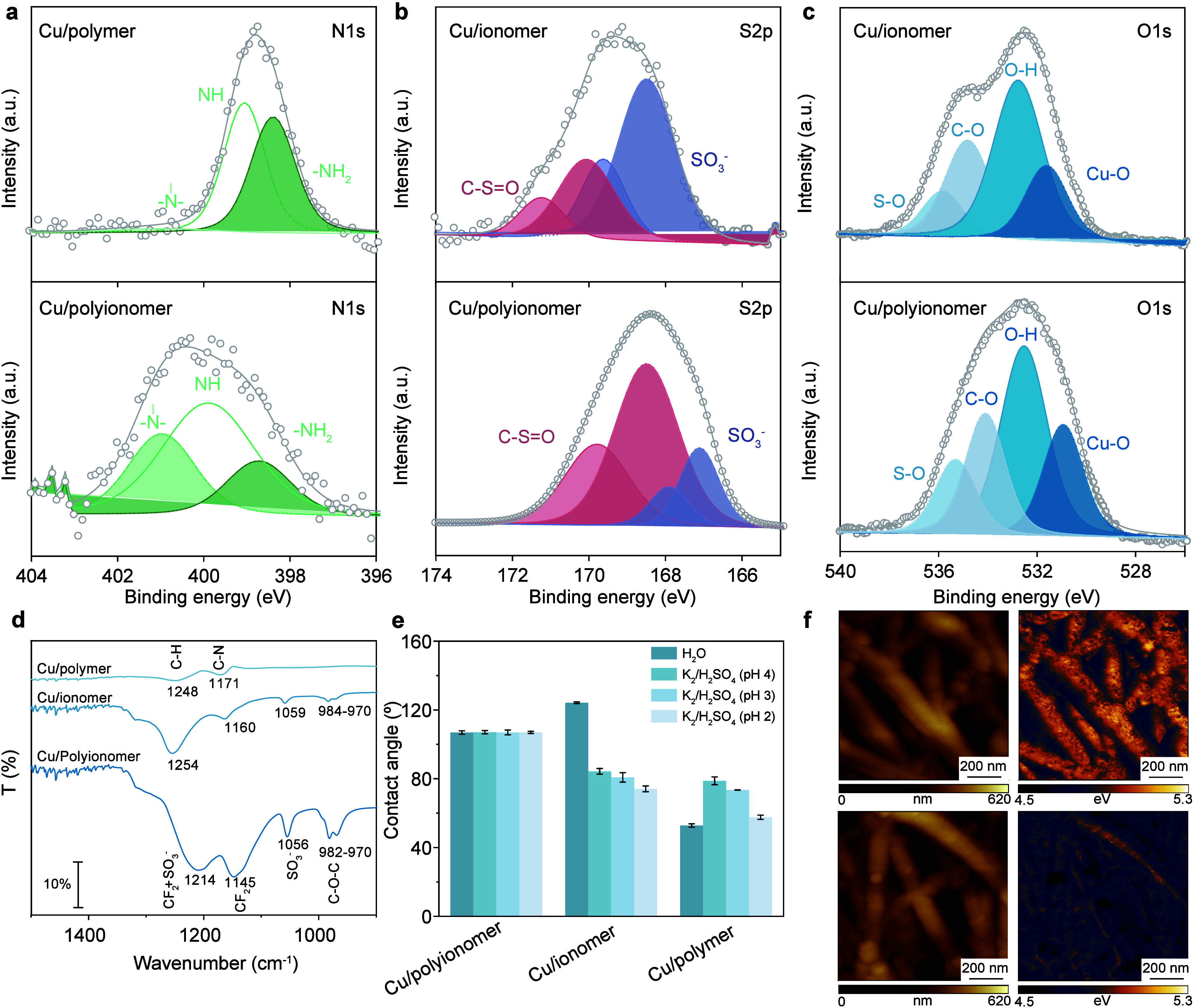
Polyionomer coated electrode characterization. Changes in electron charge density and chemical environment suggest the protonation of amine groups and interaction with sulfonic groups (a) N 1s binding energies shift to higher binding energies when comparing Cu/polyionomer with Cu/polymer. (b) S 2p shift to lower binding energies when comparing Cu/polyionomer with Cu/ionomer. (c) O 1s shift to lower binding energies when comparing Cu/polyionomer with Cu/ionomer. (d) ATR-FTIR of Cu/ionomer, Cu/polymer, and Cu/polyionomer characteristic bands between 1600 and 900 cm^–1^. Characteristic peaks for Cu/polymer and Cu/ionomer at 1248 cm^–1^ (C–H), 1171 cm^–1^ (C–N), 1254 cm^–1^ (CF_2_ + SO_3_^–^) and 1160 cm^–1^ (CF_2_) shift to 1214 cm^–1^ and 1145 cm^–1^ in the polyionomer sample. (e) Contact angle comparison in a wide pH range for Cu/polymer, Cu/ionomer and Cu/polyionomer. (f) Representative topography with respective work function maps highlighting the changes in the surface potential between Cu/ionomer (top) and Cu/polyionomer (bottom).

Shifts in the positions of XPS peaks can relate to interactions occurring due to changes in the electron density and chemical environment. We observed higher binding energies in N 1s XPS peaks in the Cu/polyionomer compared to the bare Cu/polymer coatings and lower binding energies in the Cu/polyionomer compared to the bare Cu/ionomer coatings (Figure 2a and Tables S1–S3). Concurrently, lower binding energy shifts are observed in S 2p (directly) and O 1s (indirectly) XPS peaks in Cu/polyionomer, when compared to Cu/ionomer (Figure 2b,c). These complementary shifts suggest the electronic and chemical interaction between PEI and PFSA as a form of proton scavenging, transfer of electronic charge density, and H-bonding when assembled on Cu/PTFECu. We observe a similar trend in ATR-FTIR spectra (Figure 2d), showing in Cu/polyionomer shifts in characteristic peaks of perfluorosulfonic groups and carbon–nitrogen bonds.^9,45–47^

To gain more insights into the polyionomer function, we performed contact angle measurements (Figure 2e). The similar contact angle across a wide pH range suggests that the polyionomer most exposed function (and structure) does not change drastically with pH,^48^ as opposed to individual PFSA and PEI contact angle trends, remaining comparable to the initial contact angle of PFSA at high pH. This indicates that the resulting structure could take better advantage of the hydrophobic channels in Cu/ionomer to offset [CO_2_]/[H_2_O] balance, as opposed to overexpressing the hydrophilic channels of branched polyamines.^30^

To assess the impact of the polyionomer on the electrostatic environment,^49,50^ we measured the work function combined with atomic force microscopy of the coated gas diffusion electrodes. Cu/polyionomer (4.60 ± 0.05 eV) showed a decrease in work function compared with Cu/ionomer (4.85 ± 0.1 eV) (Figure 2f). This shift, combined with the charge re-equilibration observed in XPS and FTIR, further suggests the modification of the ionomer arrangement and chemical function.

To understand the role of polyionomer in controlling the electrode–electrolyte microenvironment, we performed in situ surface-enhanced Raman spectroscopy (SERS) in the region of −0.26 to −1.46 V vs RHE in two regions: R_1_ (100–1250 cm^–1^), highlighting Cu-*CO and *C-intermediates, and R_2_ (1300–2250 cm^–1^), highlighting *CO intermediates (Figure 3a).^51,52^

**Figure 3:**
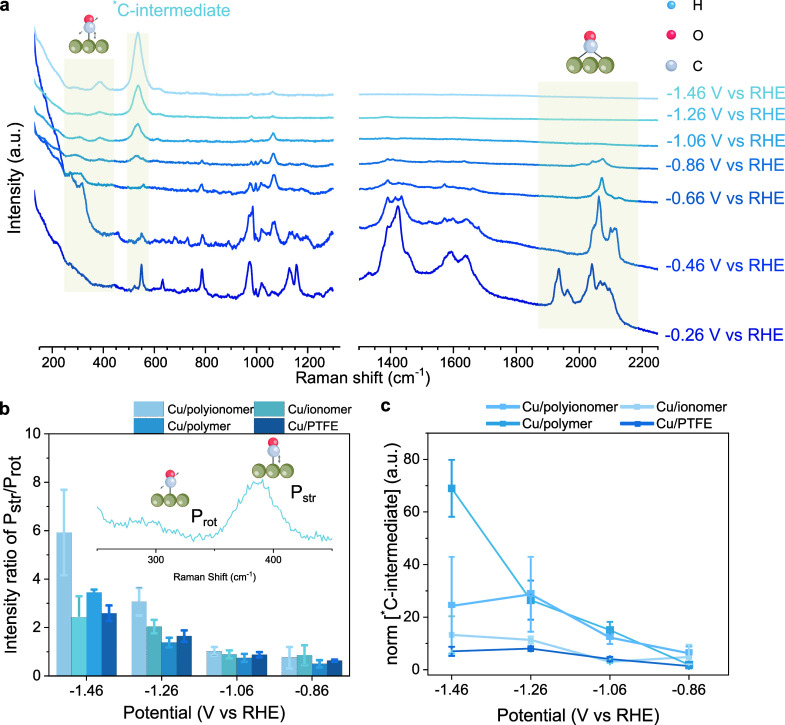
In situ Raman spectroscopy studies. (a) Raman spectrum of Cu/polyionomer sample in different applied potentials (V vs RHE) labeling corresponding intermediates or species, highlighting *CO and *C-intermediates. Samples were taken in 0.5 M K_2_SO_4_ (pH 2) in a flow cell under working potentials. (b) *CO coverage potential dependence correlation with the intensity ratio *P*_str_/*P*_rot_ of Cu/PTFE, Cu/ionomer, Cu/polymer, and Cu/polyionomer. (c) Normalized *C-intermediate area comparison between Cu/PTFE, Cu/ionomer, Cu/polymer, and Cu/polyionomer.

The intensity ratio of Cu-*CO frustrated rotation (*P*_rot_, 295–303 cm^–1^) and Cu-*CO stretching vibration (*P*_str_, 382–389 cm^–1^) often correlated with *CO coverage (Figure 3b and Figures S14–S17).^53–56^ These ratios increase for all of the catalysts, indicating that the *CO coverage increases with increasing applied potential (Figure 3b). The observed highest intensity ratio for polyionomer may indicate the highest CO_2_R activity of polyionomer among all the catalysts.

Carbon intermediate (*C-intermediate) (Figure 3c) is another important factor to directly correlate to CO_2_ reduction to C_2+_ products.^57–59^ At higher applied potential (−1.46 V vs RHE), both Cu/polyionomer and Cu/polymer show stronger *C-intermediate bands compared to Cu/ionomer and Cu/PTFE. The stabilization of *C-intermediates could be influenced by variations in electronic charge distribution and can be modulated by the presence of amine groups.^60,61^ This effect is supported by AFM/KPFM data, which suggest that the presence of PEI contributes to stabilizing this intermediate by a decrease in work function observed between the Cu/ionomer and Cu/polyionomer (Figures 2f and 3c). Further, CO_2_^–^ and *CO_2_ (Figure S15) species are observed in Cu/polymer, which may be correlated with enhanced CO_2_ adsorption and activation on active sites.^62^ This indicates that polyethylenimine (PEI) plays a crucial role in stabilizing intermediates and accelerating reaction pathways through hydrogen bonding and interactions with nearby active sites.^63^ However, these favorable thermodynamic effects alone do not translate into higher performance, as efficient CO_2_ reduction also relies on optimized reaction kinetics.^64^

To assess potential changes in kinetics derived from polyionomer coatings, we tracked the carbonate band (∼1062 cm^–1^) by in situ surface enhanced Raman spectroscopy. PFSA polymers have demonstrated improved performance in acidic media by accumulating alkali cations near the catalyst interface and buffering the local pH.^14,65,66^ The carbonate band is more intense in Cu/polyionomer compared to Cu/polymer and Cu/ionomer (Figures S14–S17) suggesting a local higher alkalinity and CO_2_ availability for Cu/polyionomer. However, due to the presence of multiple bands in this region, accurate deconvolution remains challenging.

To further understand how the polyionomer could regulate local proton activity and the reaction mechanism, we characterized selectivity at different pH and fixed current densities (Figure 4a). HER rejection and selectivity within gas products remain similar regardless of electrolyte pH for Cu/polyionomer (Figure 4b and Figure S18), whereas they vary for both ionomer and polymer samples (Figure 4a). Such pH independence is in line with contact angle measurements and could be associated with a balanced proportion of [CO_2_]/[OH^–^] (from PFSA) that compensate the mass transfer of protons to the electrode surface,^67–71^ combined with the buffering of local protons and *CO intermediate stabilization (from PEI) consistent with SERS.^72^ Consistently across all tested pH values, Cu/polyionomer electrodes require a lower potential vs SHE (standard hydrogen electrode) than either Cu/ionomer or Cu/polymer to sustain similar CO_2_R product generation (Figure S19).

**Figure 4:**
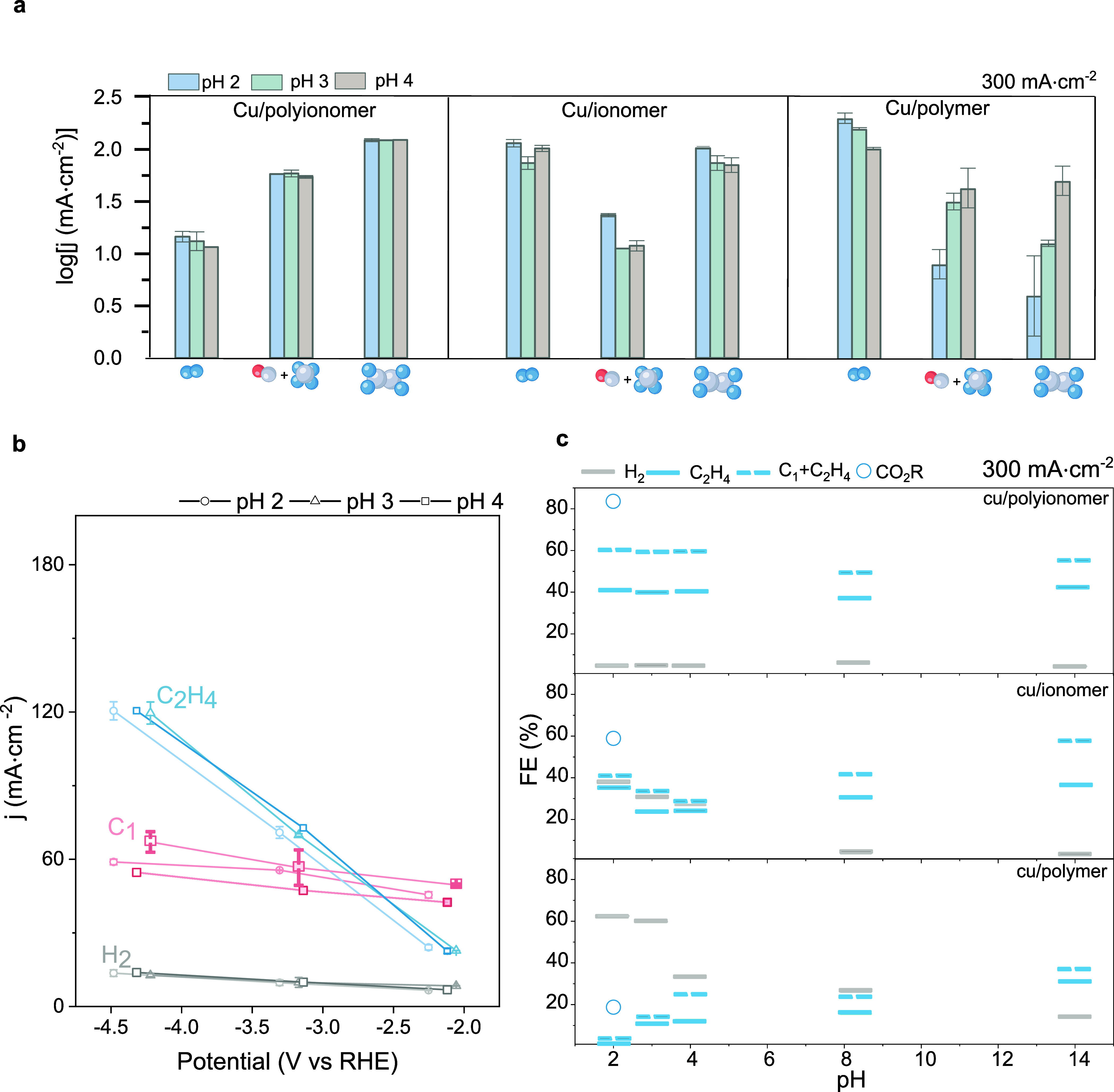
Selectivity of CO_2_ electroreduction study via electrolyte pH change. (a) Logarithm of partial current density for gas product distribution of Cu/polyionomer, Cu/ionomer, and Cu/polymer at 300 mA cm^–2^ showing pH-independence of polyionomer. (b) Cu/polyionomer partial current density of CO_2_ gas products vs potential (V vs RHE). (c) Gas product distribution at 300 mA cm^–2^ in a wider pH range up to alkaline conditions (acid, 0.5 M K_2_SO_4_: pH 2, pH 3 and pH 4; neutral, 1 M KHCO_3_; alkaline, 1 M KOH) and total products of CO_2_R at pH 2. Cu/polyionomer maintains C_2_H_4_ selectivity comparable to that of Cu/ionomer under alkaline conditions but outperforms it as pH decreases, unlike Cu/ionomer and Cu/polymer, which exhibit increased HER and reduced C–C coupling in acidic environments. This supports the suggested distinctive character of the polyionomer in preserving PFSA functionality while enabling local [H^+^] and CO regulation through PEI, thereby facilitating C–C coupling. FE (%) denotes Faradaic efficiency.

To interrogate polyionomer function, we studied gas product distribution in a wider pH range up to alkaline conditions (Figure 4c and Figure S2). Cu/polyionomer electrodes exhibit a similar H_2_ rejection and C–C coupling across the 2–14 pH range (Figure 4c and Figure S2). Cu/polyionomer shows selectivity comparable to that of the single-function Cu/ionomer at high pH. The latter shows, on the other hand, increasing HER and reducing C–C coupling as the pH decreases into the acid regime. Cu/polymer exhibits a trend similar to that of Cu/ionomer with exacerbated HER and lowered C–C selectivity. This supports the suggested distinctive character of the polyionomer retaining PFSA function and further allowing control over [H^+^] and *CO to promote C–C coupling via PEI.

To further assess the generalizability of this strategy, we performed similar electrochemical performance tests at different pH levels by using PTFE/Ag electrodes. These also reveal a pH-independent trend in Ag/polyionomer electrodes compared to Ag/ionomer electrodes (Figure S20).

We assessed the electrochemical performance of Cu/polyionomer electrodes in a flow cell at different current densities and pH 2 (Figure 5a). The maximum ethylene Faradaic efficiency was 40.1% (±0.8), with a combined C_2+_ selectivity of ∼61% at 0.3 A cm^–2^. This represents a 14% increase in C_2_H_4_ FE and approximately a 20% increase in combined C_2+_ selectivity relative to that of conventional Cu/ionomer. A maximum C_2_H_4_ partial current density of 139.5 mA cm^–2^ (*J*_total_ = 400 mA cm^–2^) is obtained for Cu/polyionomer vs 116 mA cm^–2^ for Cu/ionomer (Figure 5b and Figure S21).

**Figure 5:**
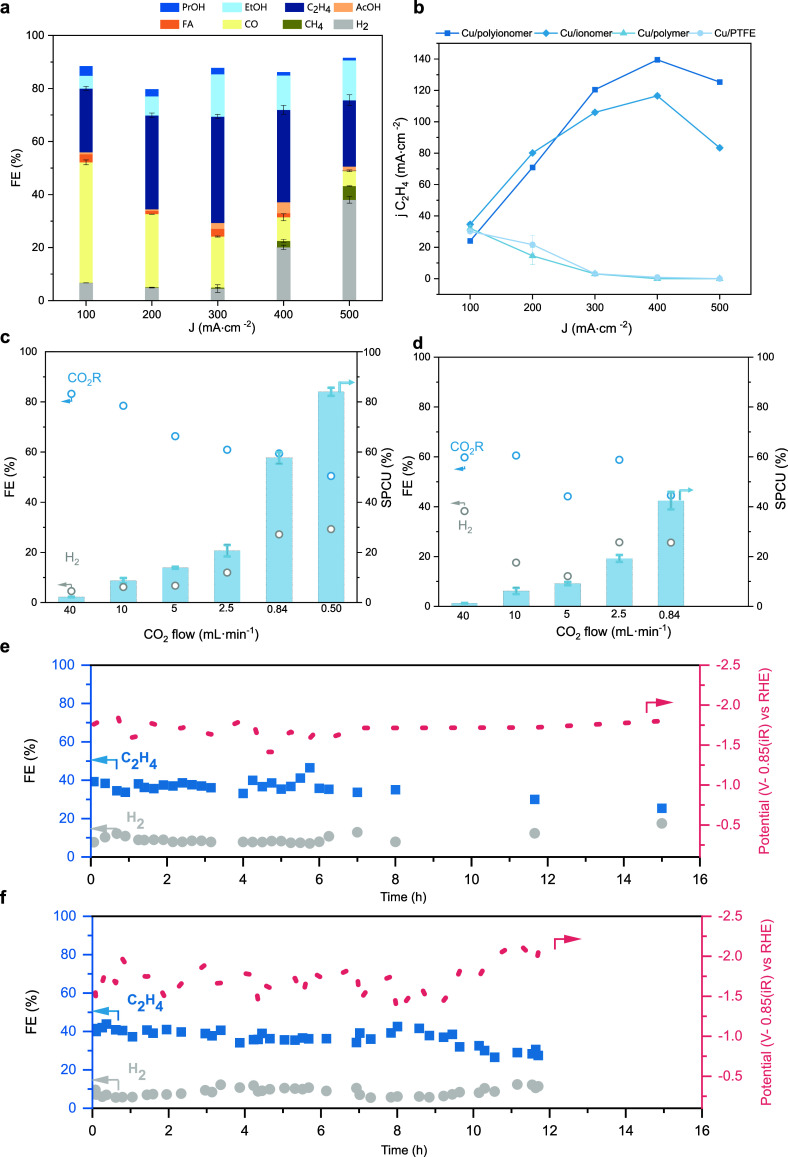
Electrocatalytic performance in 0.5 M H_2_/K_2_SO_4_(pH 2) in a flow cell. (a) Product distribution of Cu/polyionomer in the current range 0.1–0.5 A cm^–2^. FE (%) denotes Faradaic efficiency. Values are means, and error bars indicate SD (*n* = 3 replicates). (b) Ethylene partial current density of Cu/polyionomer compared with controls (Cu, Cu/ionomer, and Cu/polymer). (c) Single pass conversion of CO_2_ at different flow rates for Cu/polyionomer. (d) Single pass conversion of CO_2_ at different flow rates for Cu/ionomer. The SPCU results were obtained at a constant current density of 0.3 A cm^–2^ and an improvement of 35% in carbon utilization is observed when comparing Cu/polyionomer with Cu/ionomer. (e) Cu/polyionomer retains performance during 16 h of pulsed electrolysis at 0.3 A cm^–2^. (f) Continuous stability of Cu/polyionomer coated with Vulcan carbon at 0.3 A cm^–2^. The lifetime is extended to 24 h at 0.1 A cm^–2^ (Figure S24).

Single pass carbon utilization (SPCU) was performed at different flow rates at a fixed current density of 0.3 A cm^–2^ (Figure 5c). Decreasing the flow rate from 40 to 0.5 mL min^–1^ increased SPCU up to 84.0% (±1.6%) while maintaining a C_2+_ conversion of 64.8% (±4.1%). The corresponding FE evolution demonstrates stable product distribution over time (Figure S22). This represents one of the few systems achieving SPCU higher than 60% at application-relevant current densities (>0.2 A cm^–2^) (Table S5). Compared with Cu/ionomer, this represents a 35% net improvement in carbon utilization (Figure 5d).

The combined C_2+_ partial current density and C_2+_ carbon utilization (*j*_C2+_·CU), a proxy of product concentration in the outlet stream, shows an improvement of at least two times when compared with most Cu-based modified catalysts (Figure S23 and Table S5).

Under continuous operation at 0.1 A cm^–2^, Cu/polyionomer sustained a C_2_H_4_ Faradaic efficiency of 23 ± 5% for nearly 24 h (Figure S24a). At 0.3 A cm^–2^ a higher FE (37 ± 3%) is sustained for 2 h (Figure S24b). Post-mortem scanning electron microscopy/energy dispersive X-ray (SEM/EDX) analysis revealed that such performance decay correlates with localized salt precipitation (Figure S25). XPS and Raman conducted after operation confirmed that both PEI and PFSA remain on the electrode surface (Figures S26 and S27). These findings point toward salt crystallization and flooding as the main deactivation mechanism.

To extend the stability, we evaluated two complementary mitigation strategies. First, we explored pulsed electrolysis operation sequencing electrochemical reduction (on time) and chemical oxidation (off-time) as a means to reset the buildup of salt accumulation.^74–76^ This led to a 4-fold improvement in stability, maintaining C_2_H_4_ production for at least 8 h (Figure 5e).

Alternatively, we explored the incorporation of a thin Vulcan-carbon layer atop the Cu/polyionomer, a common strategy to extend lifetime in CO_2_R electrodes.^73^ The addition of such a porous conductive layer may inhibit salt buildup directly over the catalyst surface, which in our case resulted in extended lifetime from ∼2 to ∼10 h of continuous electrolysis with similar FEs at 0.3 A cm^–2^ (Figure 5f and Figure S24b). This performance aligns with reported systems operating under acidic conditions at high current densities, where electrode lifetimes are generally limited and most systems demonstrate stability for 10 h or less (Table S5).

## Conclusions

The electrochemical conversion of CO_2_ to multicarbon products requires the combined advance in performance metrics that enable full process viability when considering CO_2_ sourcing and product management (separation, purification, concentration). This is largely determined by the overall energy and carbon intensity, impacting costs and the environmental viability prospects. CO_2_R in acid media is one strategy that could potentially overcome energy and carbon efficiency bottlenecks but is challenged by the competition with the hydrogen evolution reaction, and insufficient energy efficiency and stability.

We demonstrate a strategy that addresses selectivity and carbon utilization in acidic media based on catalyst environment control. We found that conventional environment manipulation based on PFSA ionomers such as Nafion resulted in a pH-sensitive structure–chemical function. This was evidenced by the change in hydrophobicity and carbon vs hydrogen selectivity with lowering pH. We then designed a polyionomer coating combining PFSA and polyethylenimine that, through the interaction of their functional groups, was able to lock chemical function down to lower pH, whose function is enhanced in acidic conditions. Using in situ Raman spectroscopy, we found that such a polyionomer coating buffers local protons and balances CO_2_R intermediates, leading to improved selectivity toward C_2+_ products: with a multicarbon Faradaic efficiency of 61% and single-pass CO_2_ utilization of 84%, including a conversion efficiency of 64% toward C_2+_ at a current density of 0.3 A cm^–2^. This represents a net increase of nearly 30% in C_2+_ selectivity and 35% in carbon utilization compared to benchmark Cu/ionomer controls. The strategy presented herein offers new design handles to manipulate reaction microenvironments in electrochemical reactions and provides new ideas for future studies on the role of polyionomer structure in gas transport mechanisms by modeling atomic-level interactions over time to explain the experimentally observed improved selectivity and proton scavenging effect. Further progress would need to address the still prominent salt accumulation due to local OH^–^ formation and highly potassium concentrated electrolytes, leading to limited stability and energy efficiency. While the polyionomer strategy presented herein effectively controls proton availability and enhances C–C coupling, it does not resolve stability issues, underscoring the need for future work focused on extending operational lifetime alongside maintaining high selectivity and energy efficiency.

## Methods

### Gas Diffusion Electrode Preparation

Cu electrodes, with 300 nm thickness, were prepared by sputtering pure Cu on top of a PTFE gas diffusion layer with a 450 nm pore size. Cu/ionomer, Cu/polymer, and Cu/polyionomer were fabricated by spray coating. For a 1:10 ratio of polymer:ionomer, the loading of Nafion (PFSA) (5 wt %, Sigma-Aldrich) was 10 μL cm^–2^ and PEI (0.28 μM aqueous solution, branched, Mw 25,000) was 1 μL cm^–2^. They were dispersed in methanol (99.9%, Scharlau) and sonicated for at least 30 min before spray coating.

### Gas Diffusion Electrode Characterization

*Contact angle*: measurements were performed using the sessile drop method on a video-based contact angle system (OCA 15EC). Data were collected in 3 different spots to get an average and standard deviation. *Attenuated total reflectance-Fourier transform infrared (ATR-FTIR)*: spectra were obtained from the electrodes by using an Agilent Cary 630 FTIR spectrometer in transmittance mode. *X-ray photoelectron spectroscopy (XPS)*: spectra were obtained by measuring the electrodes using a SPECS PHOIBOS 150. XPS data analysis and fitting were carried out using CasaXPS software. The binding energies of all peaks were corrected with respect to a C 1s peak (284.5 eV). *Scanning electron microscopy (SEM)* imaging was performed with a Zeiss Auriga Crossbeam. *Atomic Force Microscopy (AFM)* measurements were performed on a Park Systems NX10 under ambient conditions in noncontact mode. For electrical measurements, the specimens were mounted on a steel disk and electrically ground using conductive silver paste and copper tape (RS Components). *Topography and nanomechanical data*: Arrow-NCR probes (*R* = 8 nm, *k* = 42 N/m, *f* = 285 kHz) were used for the topography and nanomechanical maps. The deflection sensitivity was calibrated on a sapphire standard and the spring constant via the Sader method.^77^ *Kelvin probe force microscopy (KPFM)*: Pt-Ir coated PPP-EFM probes (*R* = 25 nm, *k* = 2.8 N/m, *f* = 75 kHz) were employed for the work function mapping. The scan rates were maintained low at 0.2–0.3 Hz, which enabled the feedback control to keep a stable scan regime, reliably tracking larger topographic features.

### Electrochemical Measurements

An electrochemical three-compartment flow cell (Figure S28) was used for all electrochemical tests. The cathode gas chamber had a volume of 2.16 cm^3^ (1.2 × 1.2 × 1.5 cm), expanding to 2.5 cm^3^ when including the outlet gas tube. The anode chamber had a volume of 1.3 cm^3^ (1.2 × 1.2 × 0.9 cm). Electrochemical measurements were conducted using an Autolab 204.s workstation connected to a current booster in galvanostatic mode. The anolyte and catholyte were separated by a Nafion membrane 117. Cu/PTFE, Cu/ionomer, Cu/polymer, and Cu/polyionomer were used as cathode in different tests (electrode area of at least 1.5 cm^2^). Ag/AgCl (3.5 M KCl) was used as a reference electrode. Electrode potentials were rescaled to the reversible hydrogen electrode (RHE) reference by

4 $$E_{RHE}=E_{Ag/AgCl}+0.223\;V+0.059\times pH$$

For all measurements the anolyte was 0.5 M H_2_SO_4_ solution, and platinum mesh was used as the counter electrode. The electrolyte flow rate was 30 mL min^–1^, achieved with a peristaltic pump. A CO_2_ flow of 40 mL min^–1^ was employed for all measurements and controlled by a mass flow controller. 0.5 M K_2_SO_4_ was used as the catholyte, and the pH was adjusted using concentrated H_2_SO_4_ and checked with a pH meter. A constant volume of 20 mL was recirculated through anode and cathode compartments using peristaltic pumps. Each current was applied for at least 13 min based on prior reports.^78^ This duration allowed for sufficient time for the liquid products to form, while also enabling a comprehensive measurement of the electrochemical response under stable conditions. SPCE was performed under a constant current density of 0.3 A cm^–2^ in a flow cell at various CO_2_ flow rates and repeated at least three times to ensure reproducibility. Following the standard methodology of similar works assessing CO_2_ electrolysis at flow rates, the outlet of the gas stream was immersed in a water reservoir to maintain a constant back pressure, preventing fluctuations that could influence mass transport or reaction kinetics. The measurement duration was adjusted in accordance with the internal volume of the gas chamber, allowing sufficient time for CO_2_ to equilibrate and fully diffuse to the catalyst surface. This ensured that the observed conversion rates were representative of steady state conditions. Corresponding FE evolution demonstrates stable product distribution over time (Figure S22). Gas flow was monitored by using a calibrated mass flow controller. Stability measurement was performed with electrochemical CO_2_ reduction at a fixed current densities of 0.1 A cm^–2^ and 0.3 A cm^–2^, monitoring products over time. To overcome flooding issues, we applied two different strategies at 0.3 A cm^–2^. (1) A layer of carbon nanoparticles (Vulcan XC72, actual loading ∼0.2 mg/cm^2^)^29^ was placed atop the Cu/polyionomer and electrolyte flow decreased to 10 mL/min. Catholyte (anolyte) was exchanged with new solution after 5 h of operation, approximately. Salt accumulated at the cathode was periodically washed with Milli-Q water and N_2_. (2) An alternating electrolysis sequence of on- and off-time (15 min) after first 1 h was used. *iR* correction was made considering the solution resistance determined using EIS in −0.3 V vs RHE. Gas products from CO_2_R were analyzed using a gas chromatograph (PerkinElmer Clarus 590) coupled with a thermal conductivity detector (TCD) and a flame ionization detector (FID). Argon was used as the carrier gas. Tests were performed in triplicate. The Faradaic efficiency was calculated via

5 $$\mathrm{Faradaic efficiency (\%, FE)}=\frac{n\times F\times V_{m}\times f_{m}}{J}\times 100$$

where *n* is the number of electrons for a given product, *F* is the Faradaic constant, *V*_m_ is the molar volume, *f*_m_ is the molar reacting gas flow rate, and *J* is the current.

Single-pass CO_2_ conversion efficiency (SPCU) of CO_2_ was calculated using the equation

6 $$\mathrm{SPCU (\%)}=\frac{\frac{j}{n\times F}\times V_{m}}{f_{m}}$$

where *j* represents the partial current density of a specific product, *n* represents the number of electrons required for the specific product, *F* represents the Faraday constant, *V*_m_ represents the molar volume, and *f*_m_ is the molar reacting gas flow rate.

Liquid products were analyzed by using ^1^H NMR (Bruker 500 MHz) spectroscopy with water suppression. We used dimethyl sulfoxide (DMSO) as the reference standard and deuterium oxide (D_2_O) as the lock solvent.

### *In Situ* Raman Spectroscopy Measurements

*In situ* Raman spectroscopy were measured by Renishaw with a custom-made in situ cell by an immersion objective (L63x) covered with PFA film using a 785 nm excitation laser equipped with 1800 I/mm grating. The samples measured were Cu/PTFE, Cu/ionomer, Cu/polymer, and Cu/polyionomer. The samples were prepared by spray coating and the loading of ionomer was 10 μL cm^–2^ and 1 μL cm^–2^ of polymer, respectively. The potentials were applied by a single channel Autolab204 potentiostat using 0.5 M K_2_SO_4_ (pH 2) as electrolyte, Pt wire as counter electrode, and Ag/AgCl (3 M KCl) as reference electrode. All the data were acquired by 0.1% laser power with 2 s of laser exposure with 30 accumulations.

## Supplementary Material



## Notes

Additional electrochemical and characterization analysis, detailed pH study results, additional *in situ* Raman spectra, CO_2_R product distribution and stability, and electrochemical flow cell setup (PDF)
