# Supplementary material for: Chemostructurally Stable Polyionomer Coatings Regulate Proton-Intermediate Landscape in Acidic CO2 Electrolysis
Source: J Am Chem Soc. 2025 Jul 29;147(31):27278–88. doi: 10.1021/jacs.5c01314 (PMC12333358; doi:10.1021/jacs.5c01314)
Supplement: Supplementary file 1 [file ja5c01314_si_001.pdf]

Chemostructurally stable polyionomer coatings regulate proton-intermediate landscape in acidic CO_2 electrolysis

Bárbara Polesso[1], Adrián Pinilla-Sánchez[1], Eman H. Ahmed[1,2], Anku Guha[1], Marinos Dimitropoulos[1], Blanca Belsa[1], Viktoria Golovanova[1], Lu Xia[1], Ranit Ram[1], Sunil Kadam[1], Aparna M. Das[1], Junmei Chen[1], Johann Osmond[1], Adam Radek Martínez[1], Melanie Micali[3], Esther Alarcón Lladó[3,4], and F. Pelayo García de Arquer[1,*]

[1]ICFO - Institut de Ciències Fotòniques, The Barcelona Institute of Science and Technology, Castelldefels (Barcelona) 08860, Spain

[2]NRC- National Research Centre, Polymers and Pigments Department, Chemical industries research institute, Advanced Materials and Nanotechnology group, Cairo 12622, Egypt

[3]Center for Nanophotonics, NWO-Institute AMOLF, Science Park 104, 1098 XG Amsterdam, The Netherlands

[4]Van't Hoff Institute for Molecular Sciences (HIMS), University of Amsterdam, 1090 GD Amsterdam, The Netherlands

*corresponding author: pelayo.garciadearquer@icfo.eu

This PDF file includes:

Supplementary Figures 1-28

Supplementary Tables 1-5

Supplementary References (1-18)

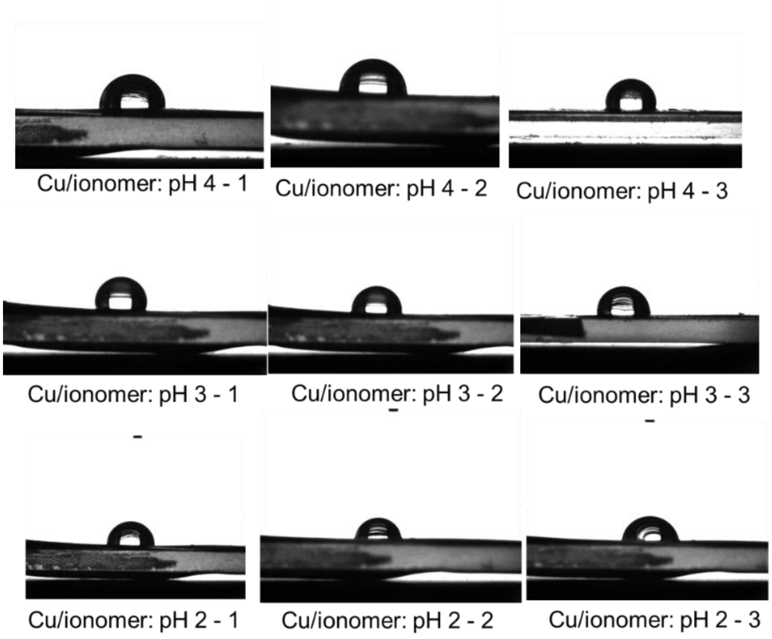

Fig. S1. Contact angle images for Cu/ionomer (PFSA) sample in different pHs. Electrolyte: 0.5 M K_2SO_4 adjusted with H_2SO_4. Measurements done with waiting time of 5 min and in different spots. Results revealed varying hydrophobicity of PFSA coatings as a function of pH.

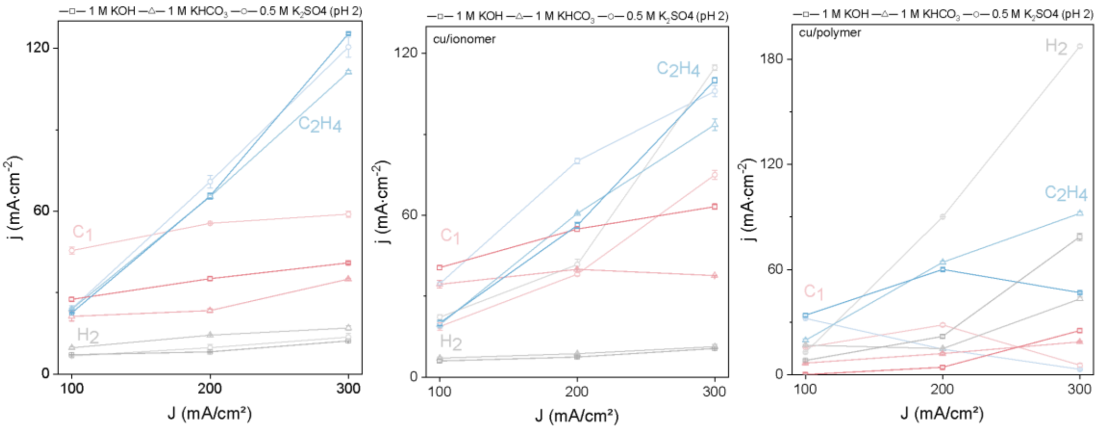

Fig. S2. Selectivity of CO_2 electroreduction study via electrolyte pH change: alkaline – 1 M KOH, neutral – 1 M $KHCO_3$ and acid – 0.5 M K_2SO_4 (pH 2). Partial current density of H_2, C_1 and C_2H_4 gas products vs current density (mA/cm²). For Cu/polyionomer in all pHs, similar product distribution is observed especially for H_2 and C_2H_4, while for Cu/ionomer and Cu/polymer, the partial current densities of C_2H_4 and C_1 products vary significantly with pH, indicating that it is strongly influenced by proton availability—either due to scarcity in alkaline conditions or excess in neutral media.

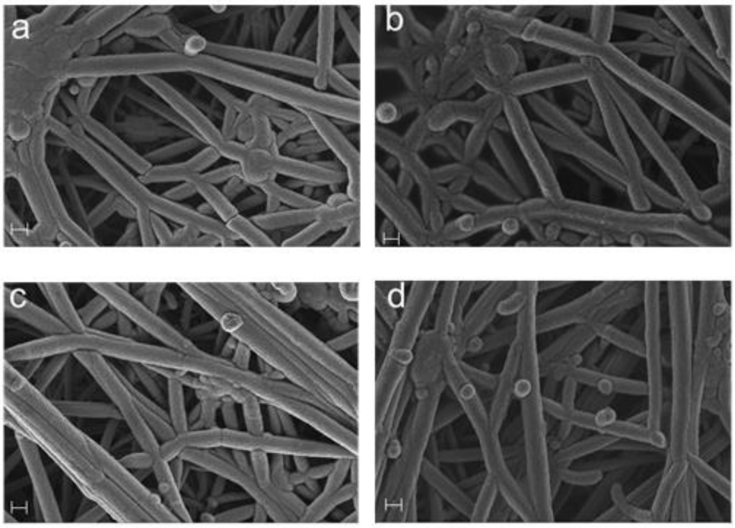

Fig. S3. SEM images of Cu-based electrode catalysts with 50 k× magnification (scale bar: 200 nm). The images were taken with an in-lens secondary electron detector. (a) Cu/PTFE; (b) Cu/ionomer; (c) Cu/polymer; and (d) Cu/polyionomer.

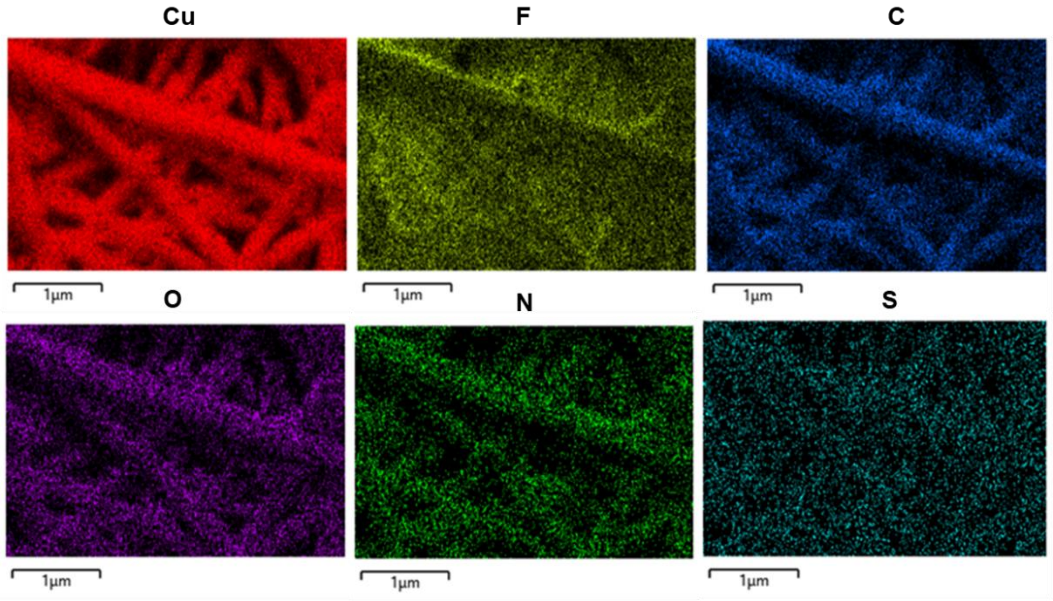

Fig. S4. EDX elemental mapping (Cu, F, C, O, N, S) of Cu/polyionomer showing homogeneous distribution.

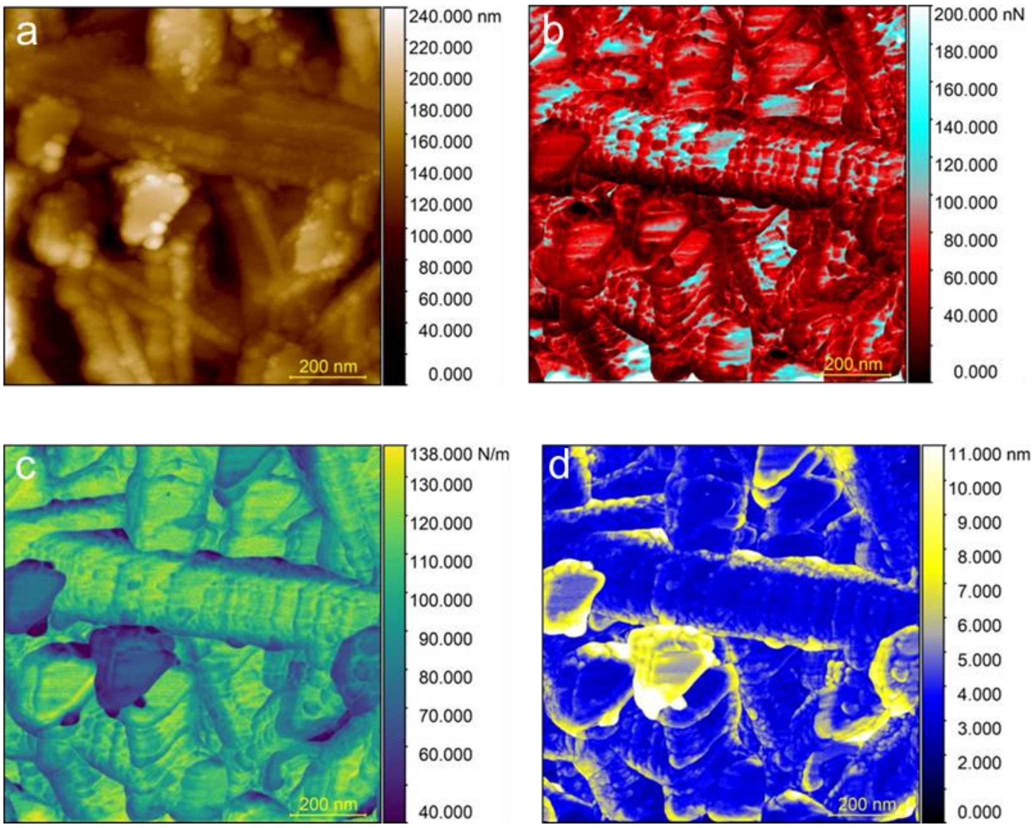

Fig. S5. Nanomechanical maps showing homogeneous distribution of Cu/polyionomer. (a) topography; (b) adhesion; (c) Young's Modulus; and (d) deformation.

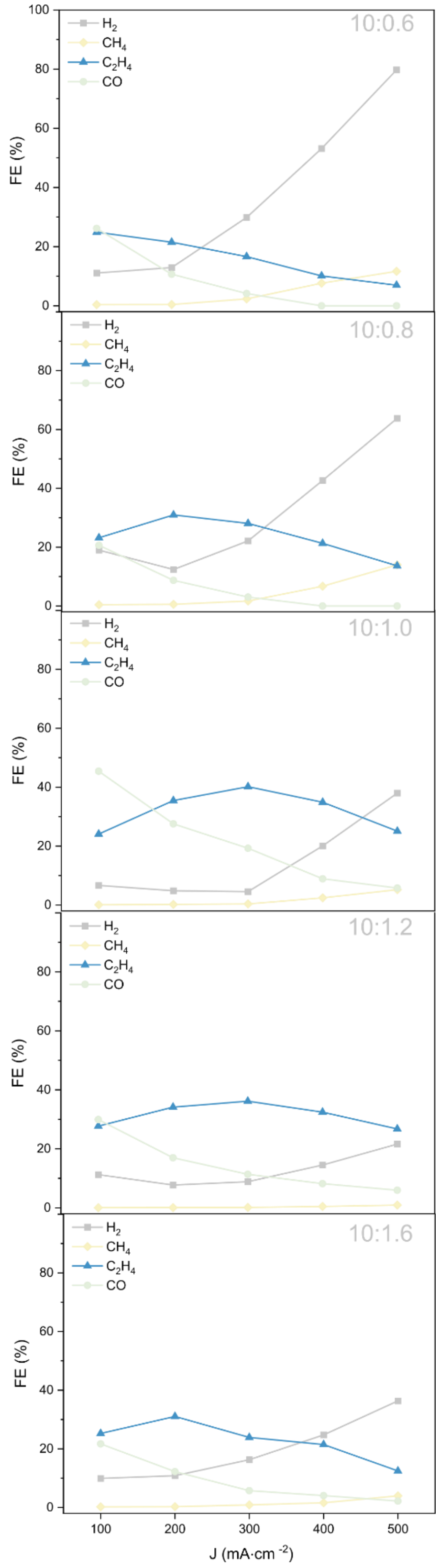

Fig. S6. Gas faradaic efficiency (FE) of Cu/polyionomer samples with different ratio of branched PEI from 100 mA·cm^{-2} to 500 mA·cm^{-2}.

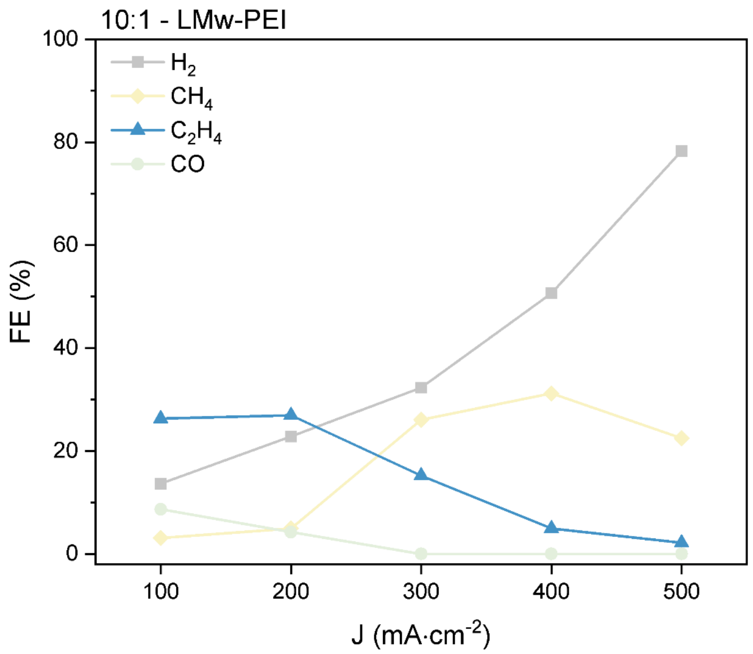

Fig. S7. Gas faradaic efficiency (FE) of Cu/polyionomer sample LMw-PEI from 100 mA·cm^{-2} to 500 mA·cm^{-2}. Sample was operated in 0.5 M K_2SO_4 (pH 2) in a flow cell.

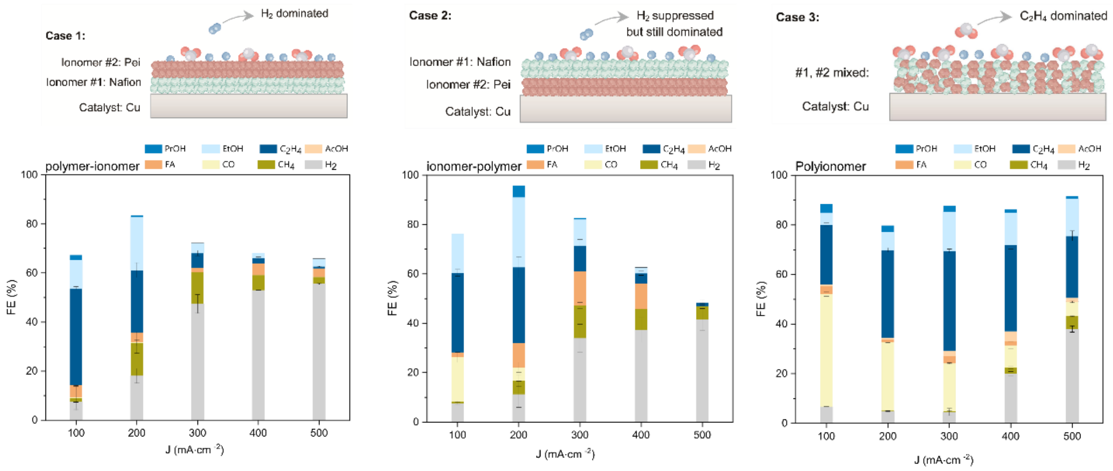

Fig. S8. Three different electrode configurations and Faradaic efficiency (FE) distribution of case: (1) bilayer polymer-ionomer; (2) bilayer ionomer-polymer; and (3) mixture polyionomer. Sample were operated in 0.5 M K_2SO_4 (pH 2) in a flow cell.

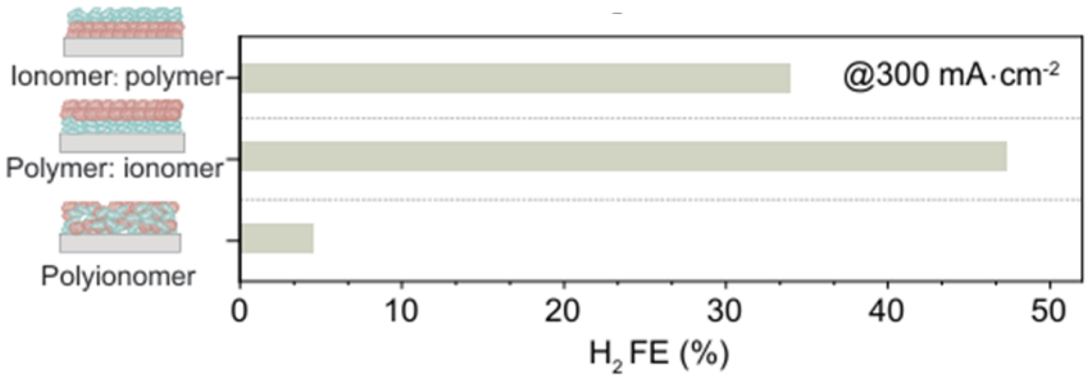

Fig. S9. Hydrogen Faradaic Efficiency (FE) at 300 mA·cm-² of different electrode configuration: bilayer (ionomer (blue):polymer (pink) and polymer (pink):ionomer (blue)) vs mixture (polyionomer).

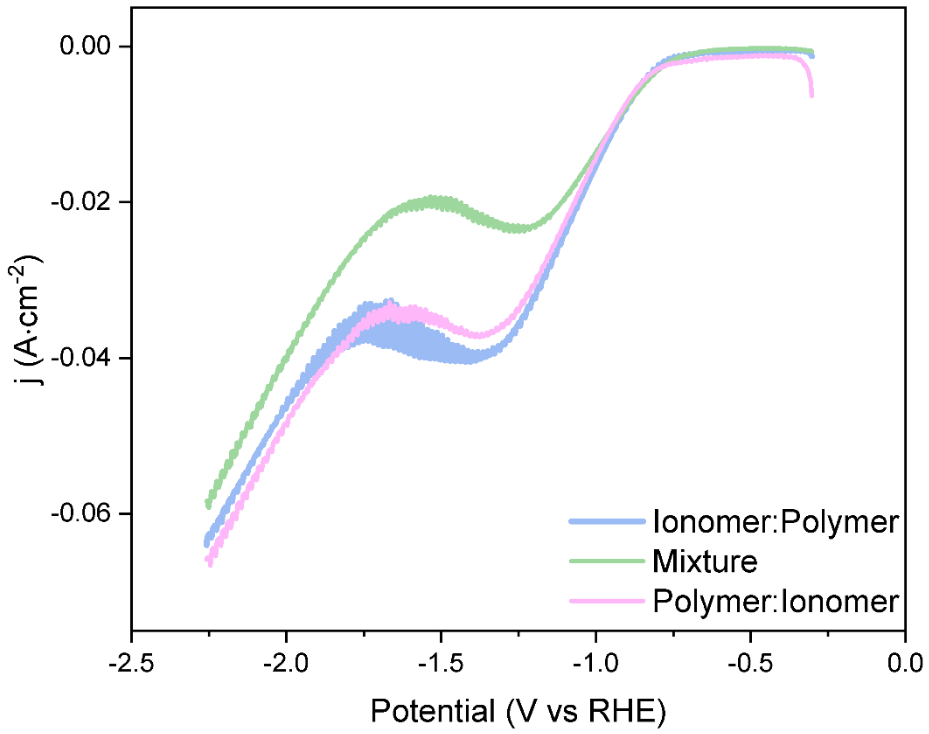

Fig. S10. LSV (Linear sweep voltammetry) curves of different polymer/ionomer configurations with Ar (40 mL·min^{-1}). Scan rate was 50 mV·s^{-1}. Sample was operated in 0.5 M K_2SO_4 (pH 2) in a flow cell.

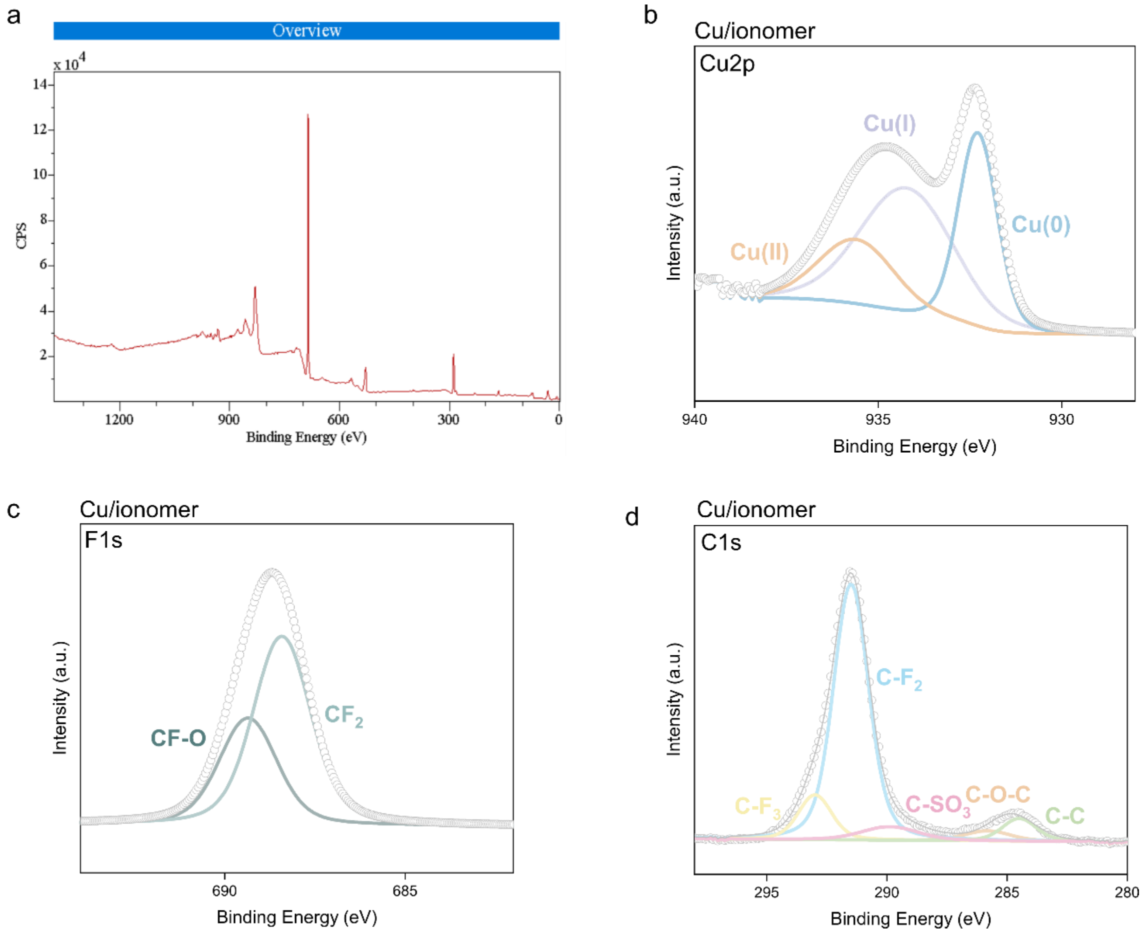

Fig. S11. XPS spectra of Cu/ionomer. (a) Survey; (b) Cu2p; (c) F1s; and (d) C1s. Spectra were obtained by measuring the electrodes using SPECS PHOIBOS 150. XPS data analysis and fitting were carried out using CasaXPS software.

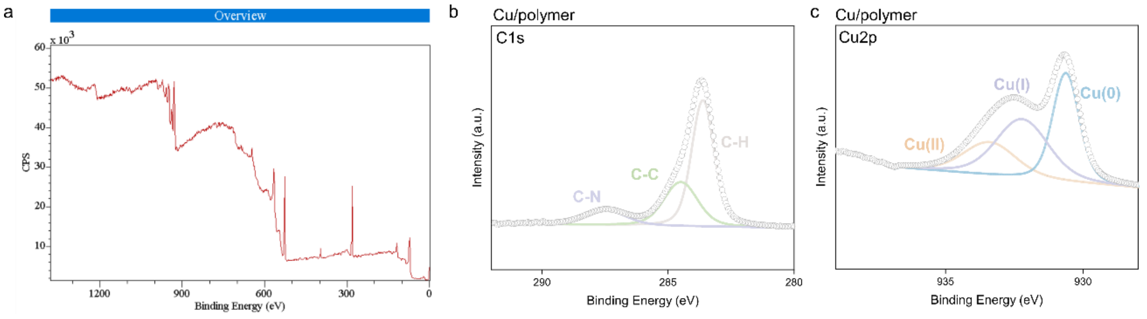

Fig. S12. XPS spectra of Cu/polymer. (a) Survey; (b) Cu2p; (c) C1s. Spectra were obtained by measuring the electrodes using SPECS PHOIBOS 150. XPS data analysis and fitting were carried out using CasaXPS software.

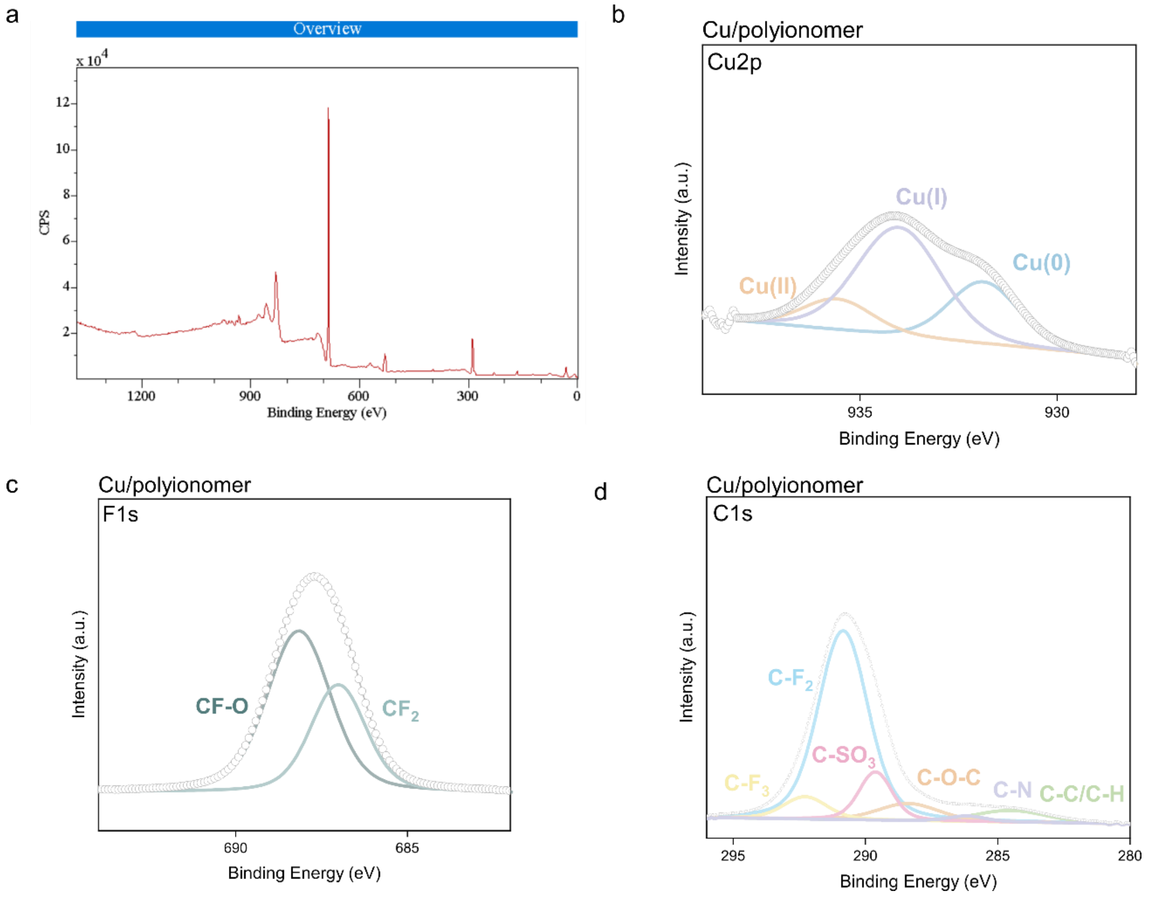

Fig. S13. XPS spectra of Cu/polyionomer. (a) Survey; (b) Cu2p; (c) F1s; and (d) C1s. Spectra were obtained by measuring the electrodes using SPECS PHOIBOS 150. XPS data analysis and fitting were carried out using CasaXPS software.

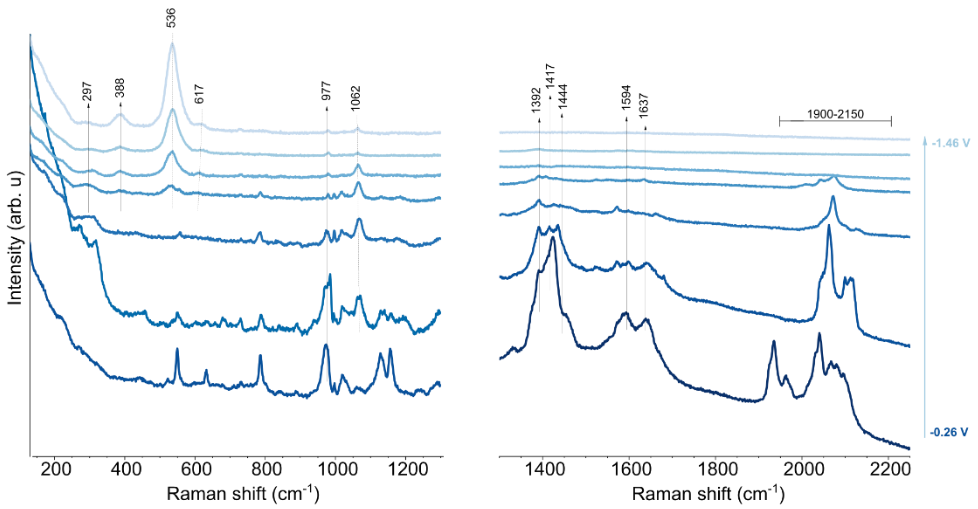

Fig. S14. *In situ* Raman spectra of Cu/polyionomer catalyst. The Raman spectrum of Cu/polyionomer sample shows the presence of specific intermediates and groups. Samples were operated in 0.5 M K_2SO_4 (pH 2) in a flow cell under working potentials. At -0.6 V vs. Ag/AgCl, carbonate bands either disappear or become too small to be resolved with increasing applied voltage, making it difficult to estimate the local pH in this way. Also, due to the presence of multiple bands in this region, accurate deconvolution remains challenging.

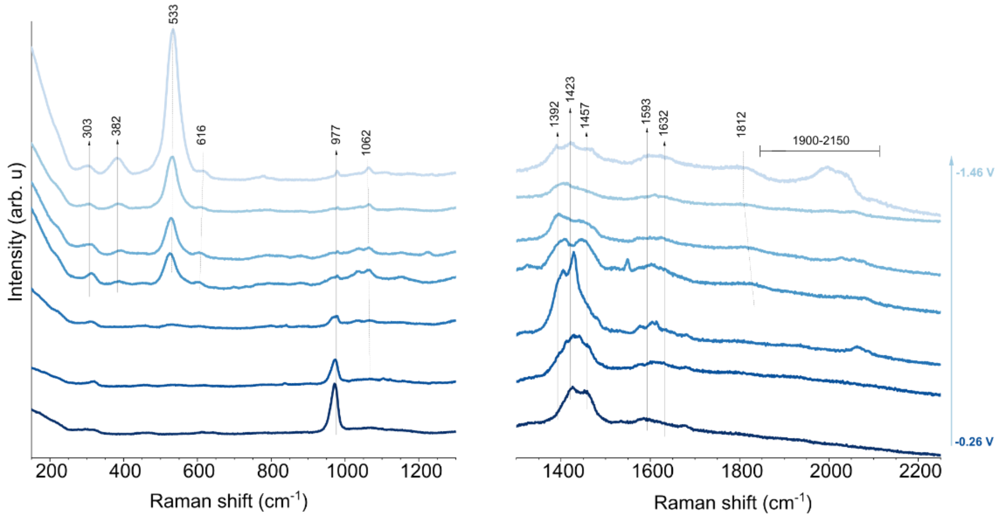

Fig. S15. *In situ* Raman spectra of Cu/polymer catalyst. The Raman spectrum of Cu/polymer sample shows the presence of specific intermediates and groups. Samples were operated in 0.5 M K_2SO_4 (pH 2) in a flow cell under working potentials.

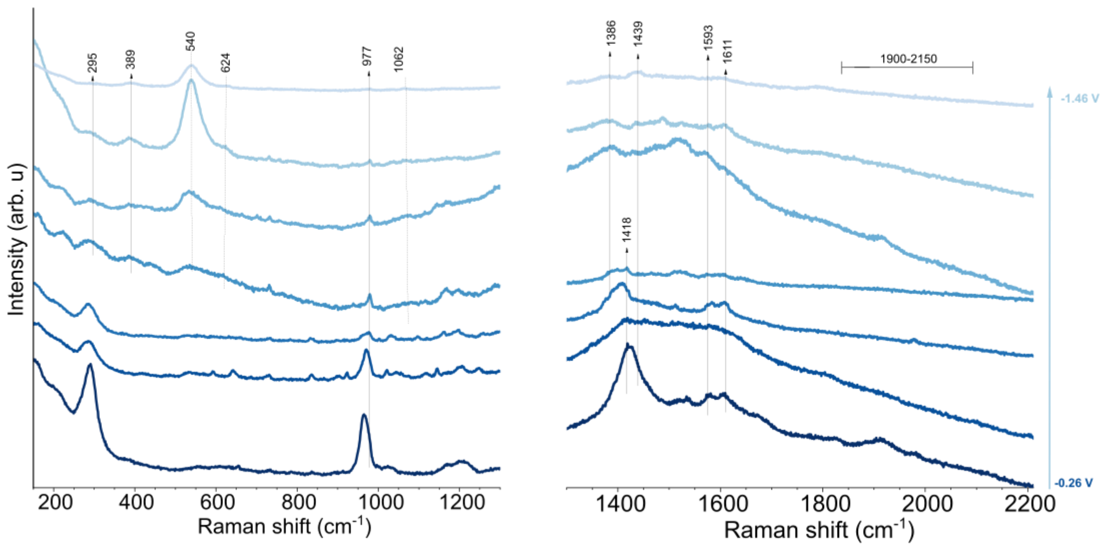

Fig. S16. *In situ* Raman spectra of Cu/ionomer catalyst. The Raman spectrum of Cu/ionomer sample shows the presence of specific intermediates and groups. Samples were operated in 0.5 M K_2SO_4 (pH 2) in a flow cell under working potentials.

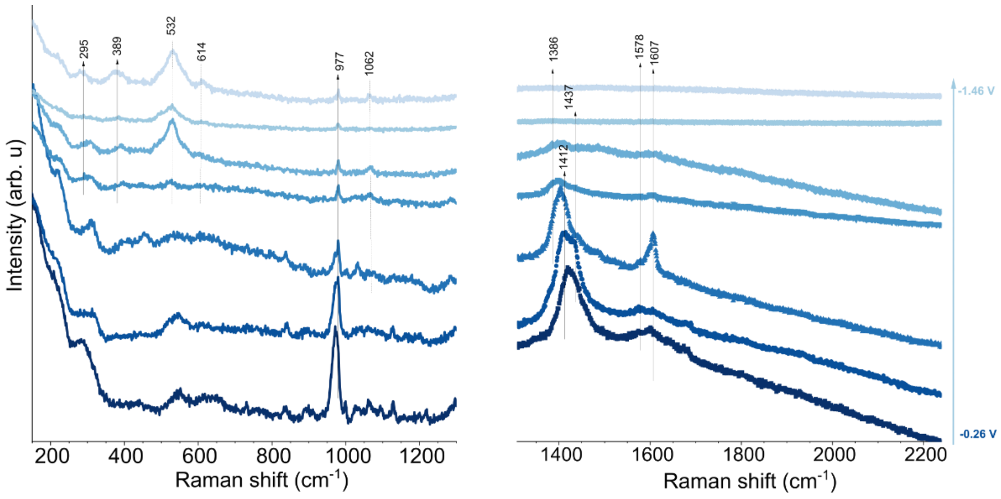

Fig. S17. *In situ* Raman spectra of Cu/PTFE catalyst. The Raman spectrum of Cu/PTFE sample shows the presence of specific intermediates and groups. Samples were operated in 0.5 M K_2SO_4 (pH 2) in a flow cell under working potentials.

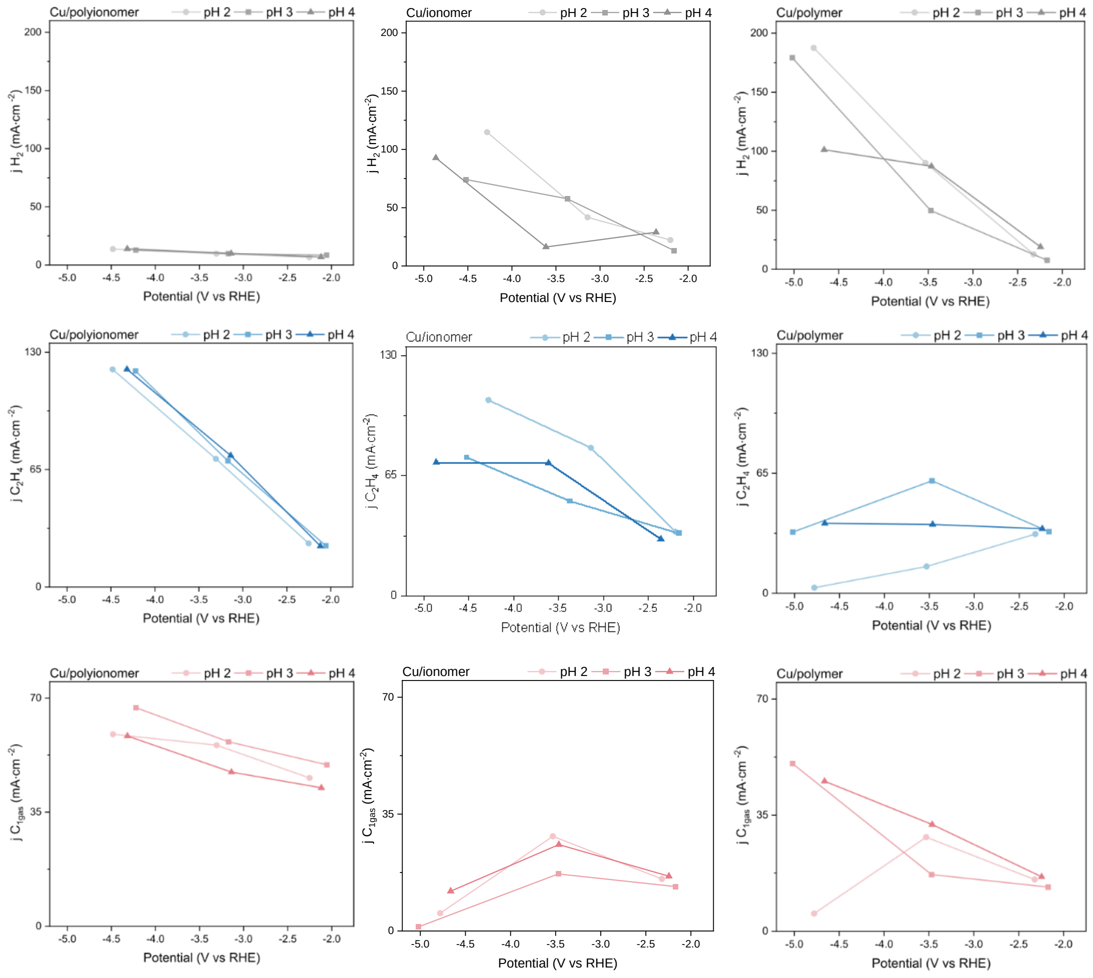

Fig. S18. Selectivity of CO_2 electroreduction study via electrolyte pH change. Cu/polyionomer partial current density of H_2, C_2H_4 and C_1 gas products vs Potential (V vs RHE) comparison with Cu/ionomer and Cu/polymer. Samples were operated in 0.5 M K_2SO_4 (Different pH) in a flow cell. pH was adjusted using concentrated H_2SO_4 and checked with a pH meter.

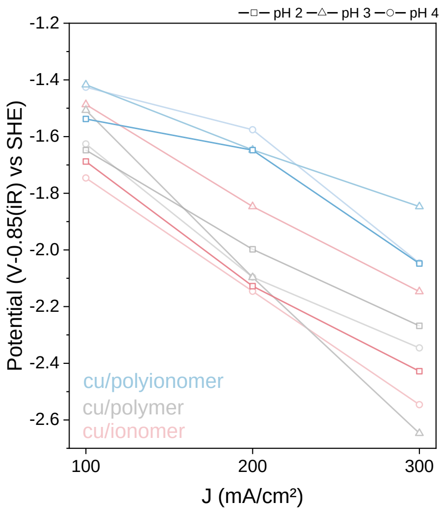

Fig. S19. Potential (V- 0.85·iR vs SHE) vs current density (mA/cm²) in different pHs. Cu/polyionomer (blue) requires a lower potential vs. SHE than either Cu/ionomer (pink) or Cu/polymer (grey) at all tested pH values. This overpotential reduction may come from the polyionomer's ability to enrich CO_2 at the catalyst interface, stabilize the H^+ supply, buffer local pH, and stabilize key $^*CO/^*COOH$ intermediates

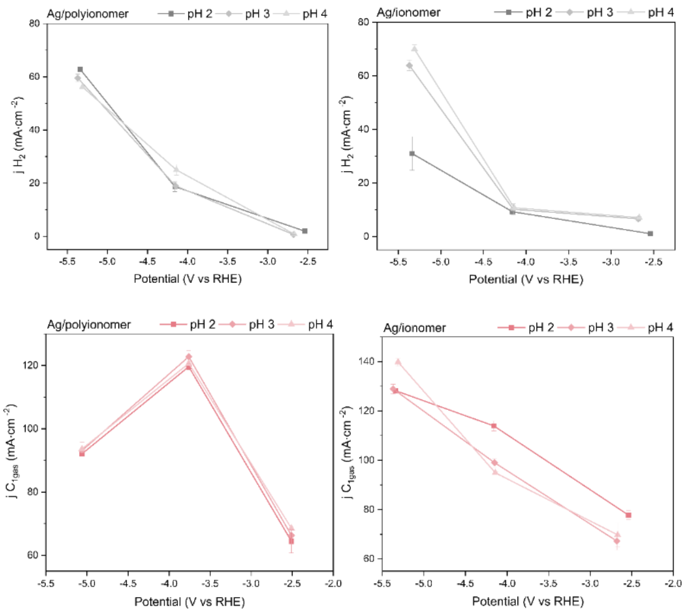

Fig. S20. Selectivity of CO_2 electroreduction study via electrolyte pH change. Ag/polyionomer partial current density of H_2, and C_1 gas products vs Potential without iR correction (V vs RHE) comparison with Ag/ionomer. Samples were operated in 0.5 M K_2SO_4 (Different pH) in a flow cell. pH was adjusted using concentrated H_2SO_4 and checked with a pH meter. The results indicate a pH-independent trend for Ag/polyionomer electrodes compared to Ag/ionomer electrodes.

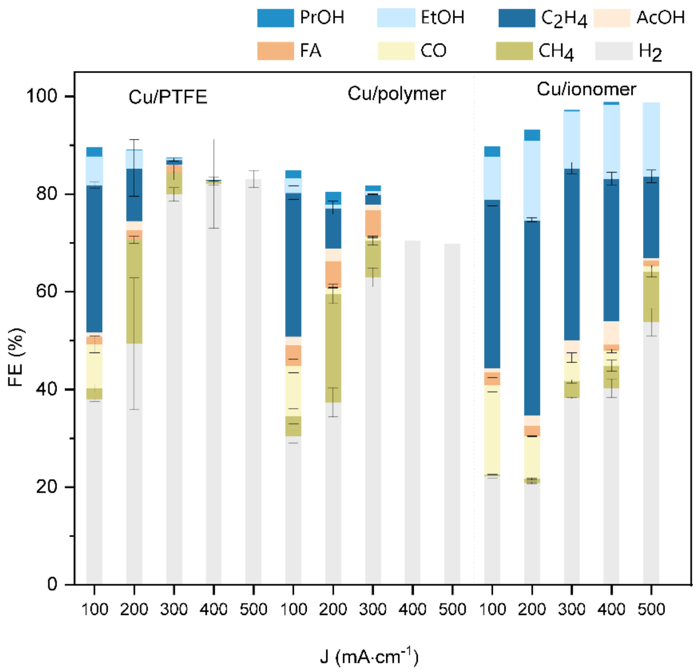

Fig. S21. Faradaic efficiency (FE) of control samples: Cu/PTFE, Cu/polymer and Cu/ionomer 100 mA·cm^{-2} to 500 mA·cm^{-2}. Values are means, and error bars indicate SD (n = 3 replicates). Samples were operated in 0.5 M K_2SO_4 (pH 2) in a flow cell.

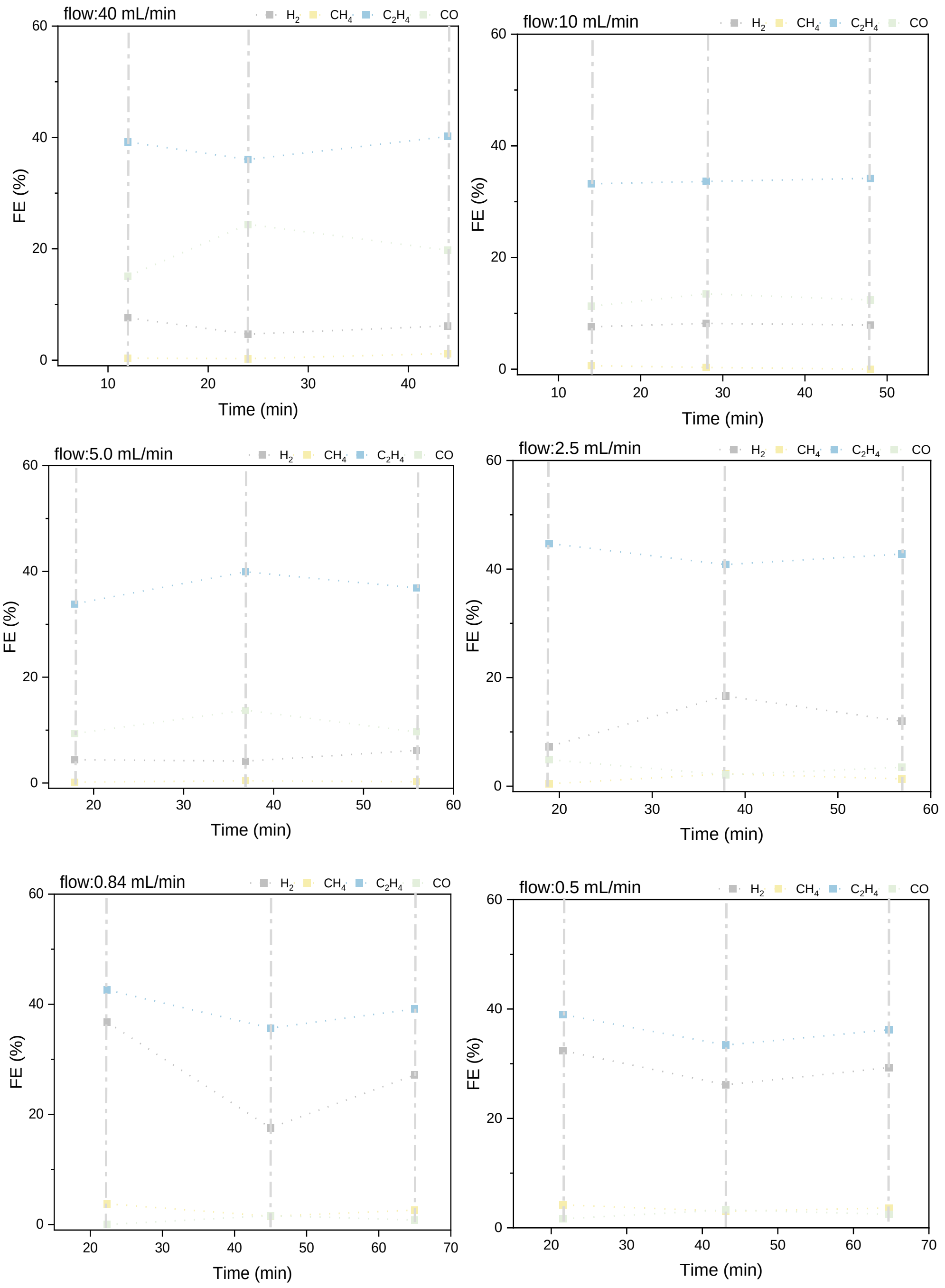

Fig. S22. Corresponding FE evolution demonstrates stable product distribution over time. FE data was used to calculate single pass conversion shown in Fig. 5c. Vertical dashed lines represent GC injection times showing when gas samples were taken during CO_2R measurement.

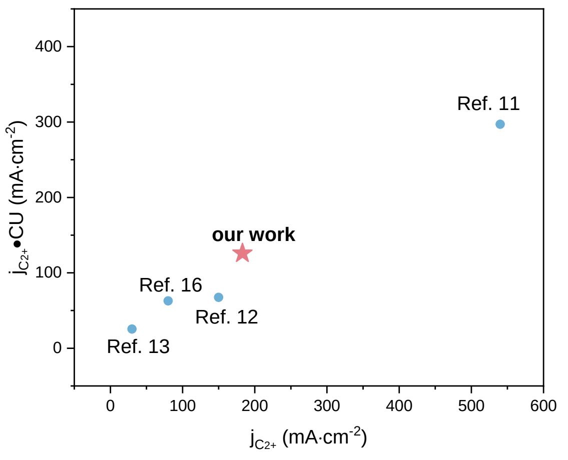

Fig. S23. Combined relevant performance metrics combined relevant performance metrics from a viability perspective (j_{C2+} x CU), our strategy improved two times most of already reported Cu-based catalyst with ionomer modifications in acidic conditions.

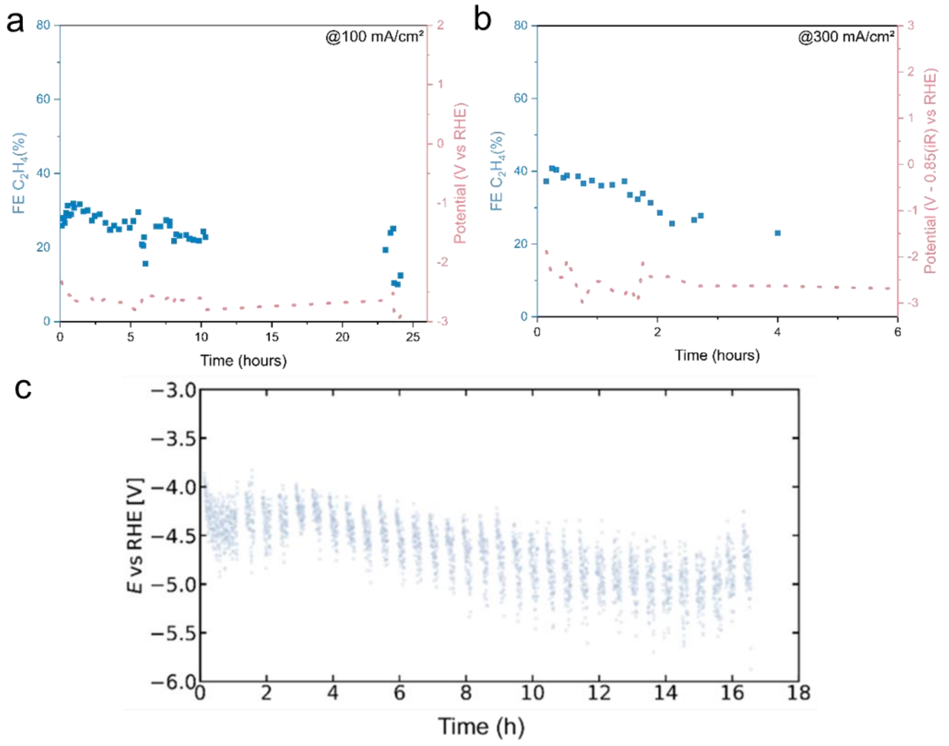

Fig. S24. Different stability tests: a) C2H4 FE (blue) and potential (red) of Cu/polyionomer at a continuous constant current density of 100. C_2H_4 FE (%) remained at around 23% (± 5%) during almost 24 hours in continuous operation at 100 mA/cm². b) C_2H_4 FE (%) remained at around 37% (± 3%) during 2 hours at 300 mA/cm². c) Recorded potential of stability test applying alternating electrolysis sequence of on- and off-time at the constant current density of 300 mA·cm^{-2} over 16 hours of continuous testing and 8 hours of active operation ("on" time). Samples were operated in a flow cell (pH 2) with 0.5 M K_2SO_4 (catholyte) and 0.5 M H_2SO_4 (anolyte).

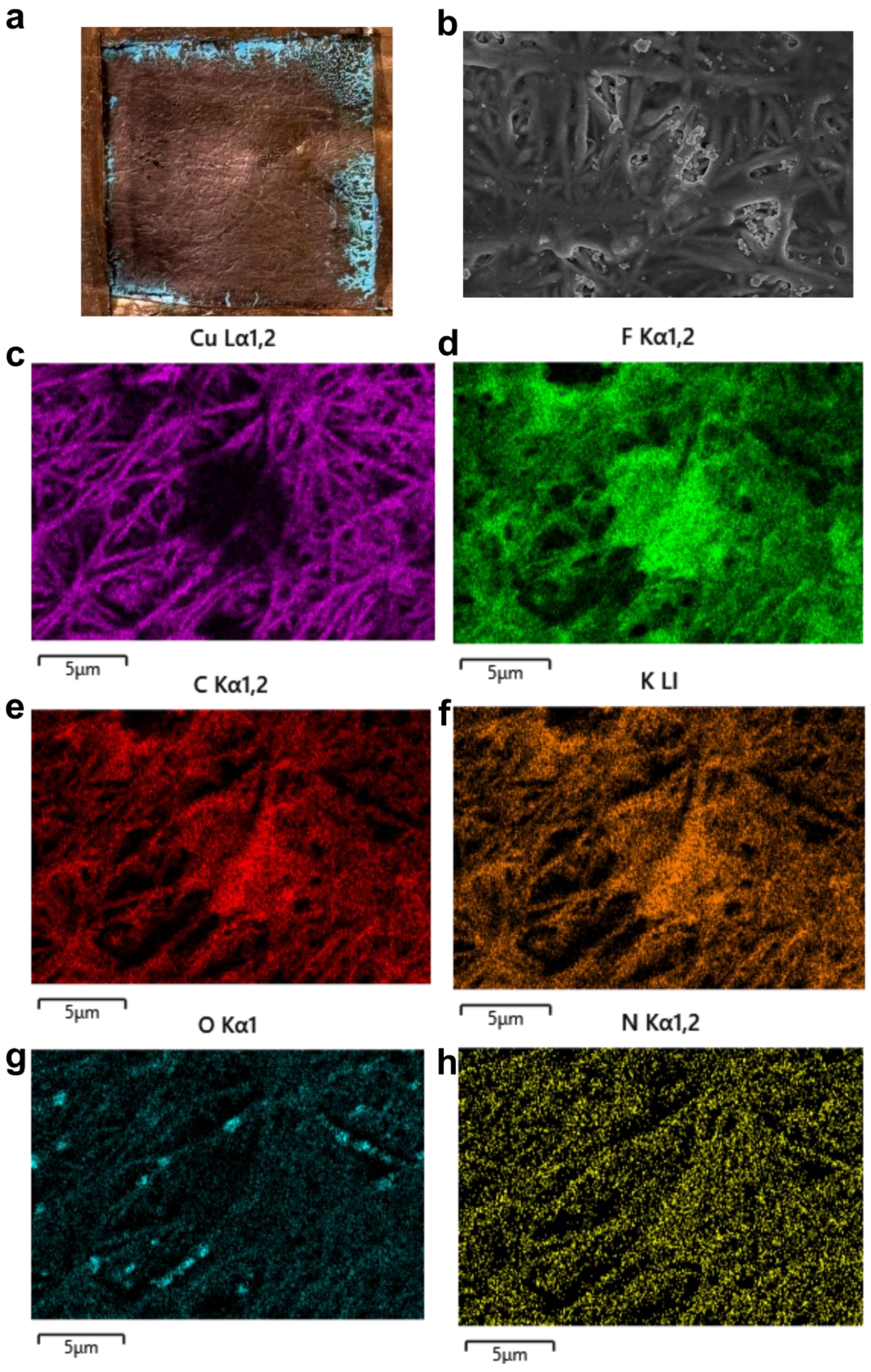

Fig. S25. Post-electrolysis analysis of the Cu/polyionomer: a) visual; b) SEM; c-h) EDX. These suggest that salt precipitation led to electrode flooding, contributing to the observed instability after 2 hours of operation

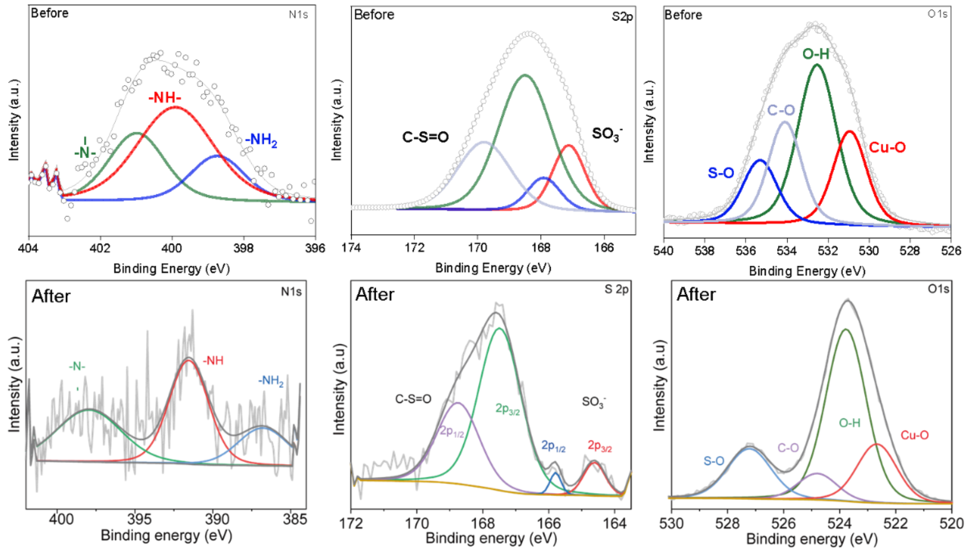

Fig. S26. XPS of Cu/polyionomer after four hours continuous stability test at 0.3 A·cm^{-2} suggesting PEI and PFSA remain present on the electrode surface. Nevertheless, the decrease in binding energies suggests potential deactivation, which could impact long-term stability.

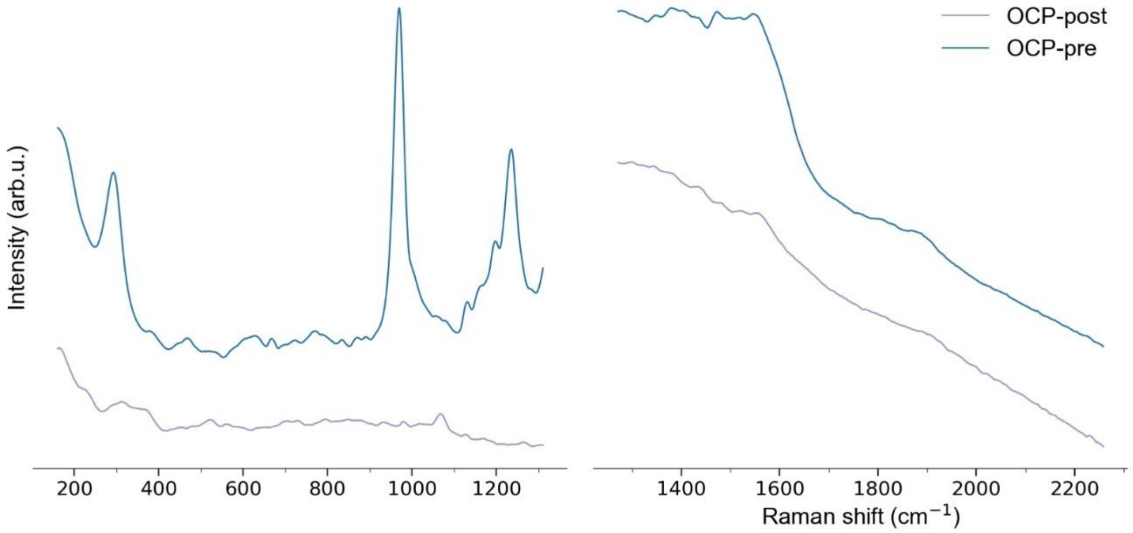

Fig. S27. OCP spectra from *In situ* Raman of Cu/polyionomer catalyst before (OCP-pre) and right after (OCP-post) electrolysis. Samples were operated in 0.5 M K_2SO_4 (pH 2) in a flow cell. Minimal differences are observed, with the primary features remaining unchanged. Before CO_2R and after CO_2R, a peak associated with surface O_2CO (bidentate carbonate) is observed, along with a smaller peak around 1070 cm^{-1}, both indicative of carbonate species. Disappearance of SOx at 970 cm^{-1} and C-F (1100-1300 cm^{-1}) after CO_2R, may indicate Cu/polyionomer rearrangement.

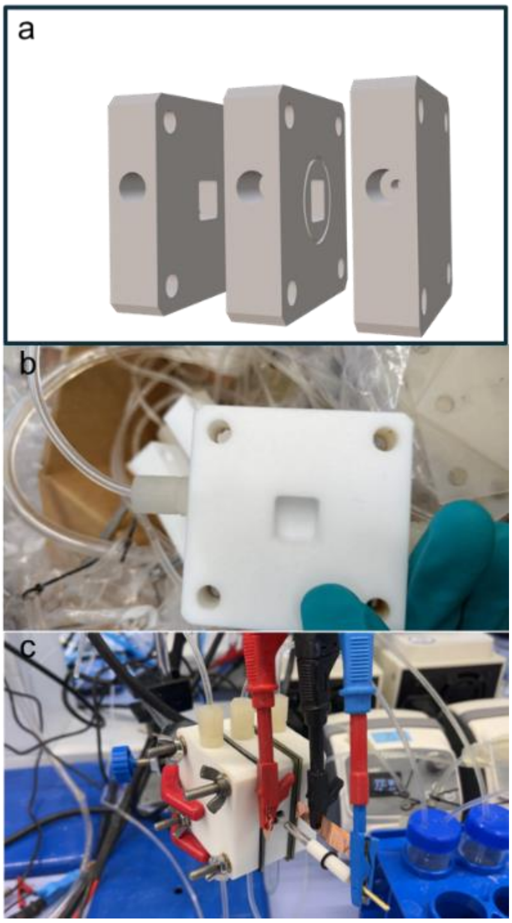

Fig. S28. Electrochemical flow cell: a) gas diffusion flow cell with dimensions (cathode chamber: 1.2 x 1.2 x 1.5 cm; anode chamber: 1.2 x 1.2 x 0.9 cm; middle compartment: 1.2 x 1.2 cm); b) cathode chamber; c) full set-up.

Table S1. XPS peak fitting and composition for Cu-ionomer[1–4]. The binding energy of all peaks were corrected with respect to C1s peak (284.5 eV).

C1s	eV	At. (%)
C-C	284.5	6.16
C-O-C	285.8	3.63
C-SO_3	289.9	6.12
CF_2	291.5	72.2
CF_3	293.0	11.9
**Cu2p**	**eV**	**At. (%)**
Cu(0)	932.3	31.43
Cu(I)	934.2	48.22
Cu(II)	935.6	20.35
**F1s**	**eV**	**At. (%)**
O-CF	689.4	36.13
CF_2	688.4	63.87
**S2p**	**eV**	**At. (%)**
SO_3^-		
2p 3/2	168.5	47.79
2p ½	169.6	18.89
C-S=O		
2p 3/2	170.1	23.88
2p ½	171.2	9.44
**O1s**	**eV**	**At. (%)**
Cu-O	531.6	18.35
O-H	532.7	45.39
C-O	534.8	25.30
SO_3	535.8	10.96

Table S2. XPS peak fitting and composition for Cu-Polymer[1–4]. The binding energy of all peaks were corrected with respect to C1s peak (284.5 eV).

C1s	eV	At. (%)
C-H	283.6	57.92
C-C	284.5	29.93
C-N	287.5	12.16
**Cu2p**	**eV**	**At. (%)**
Cu(0)	930.6	37.62
Cu(I)	932.2	40.00
Cu(II)	933.4	22.39
**N1s**	**eV**	**At. (%)**
NH_2	398.4	45.33
N-H	399.1	50.22
-N-	400.6	4.42

Table S3. XPS peak fitting and composition for Cu/polyionomer[1–4]. The binding energy of all peaks were corrected with respect to C1s peak (284.5 eV).

C1s	eV	At. (%)
C-C/C-H	284.5	5.82
C-N	286.2	1.45
C-O-C	288.4	7.31
C-SO_3	289.6	11.36
CF_2	290.8	67.11
CF_3	292.3	6.94
**Cu2p**	**eV**	**At. (%)**
Cu(0)	931.9	27.54
Cu(I)	934.0	58.74
Cu(II)	935.6	13.72
**F1s**	**eV**	**At. (%)**
O-CF	687.0	36.68
CF_2	688.2	63.32
**S2p**	**eV**	**At. (%)**
SO_3^-		
2p 3/2	167.1	15.59
2p ½	167.9	7.79
C-S=O		
2p 3/2	168.5	51.09
2p ½	169.8	25.53
**O1s**	**eV**	**At. (%)**
Cu-O	530.9	21.06
O-H	532.5	41.55
C-O	534.1	22.80
SO_3	535.3	14.59
**N1s**	**eV**	**At. (%)**
NH_2	398.7	18.46
N-H	399.9	53.30
-N-	400.9	28.24

Table S4. In situ Raman wavenumber and corresponding intermediate species [5–10].

Wave number (cm^{-1})	Correspondence
295 – 303	Cu-CO_{rot}
382 – 389	Cu-CO_{str}
532 – 540	*C-intermediate/*OH
614 - 624	Cu-O_{ads}
977	SO_4^{2-}
1062	$CO_3^{2-}{}_{ads}$
1386 – 1392	HCO_3^-
1412 – 1423	$CO_3^{2-}{}_{free}$
1439 – 1457	CO_2^-
1578 – 1594	$CO_3^{2-}{}_{bident}$
1607 – 1637	H_2O
1812	$^*CO_2$
1900 – 2150	$^*CO$

Table S5. Performance table of state-of-art in Cu-based with binders in acid media.

Catalyst	Conditions	C_2+ FE (%)	Current density ($mA \cdot cm^{-2}$)	Jpar	SPC (%)	$Jpar^xCU$	Stability (h)	References
**Cu/polyionomer**	**0.5 M K_2SO_4 H_2SO_4 (pH=2)**	**61**	**300**	**183**	**84.0 (± 1.6) 68.8 (± 4.1) (C_{2+})**	125.9	**~10**	**This work**
CAL-modified CuNP	H_3PO_4 1M / KCl 3M (pH 1)	45	1200	540	77 55 (C_{2+})	297	12	[11]
COF:PFSA-modified PTFE–Cu	1 M H_3PO_4 + 3 M KCl (pH=1)	75	200	150	45 (C_{2+})	67.5	20	[12]
PCRL (Cu/PTFE/ionomer)	MEA	30	100	30	85 (C_{2+})	25.5	8	[13]
Cu/C (carbon black)	0.1 M H_2SO_4-K_2SO_4 (pH=1)	36	550	198	-	-	-	[14]
Modified-Cu/PTFE	1 M $H_3PO_{4\,+}$ 0.1M K^+ (pH=1)	55	50	27.5	-	-	5	[15]
Cu/benzimidazolium CG/CN/Nafion	0.2 M H₂SO4 (pH=0.4)	80	100	80	90 78.5 (C_{2+})	62.8	150	[16]
CuNP/EmimBF₄	0.05M H_2SO_4+ 3M KCl (pH= 0.89)	60	300	180	-	-	5	[17]
$Cu_{0.92}Sn_{0.08}$	3 M KCl/0.05 M H_2SO_4 (pH =1)	~65	400	260	~78 (500 $mA.cm^{-2}$)	-	-	[18]

poly(Lys, Phe)-modified EC–Cu	3~8 mM $CuSO_4$, 0.05 M H_2SO_4 and 2.5 M KCl	90	200	180	70	126	10	[8]

References

1. Li, L. *et al.* Processing, Characterization, and Impact of Nafion Thin Film on Photonic Nanowaveguides for Humidity Sensing. *Adv. Photonics Res.* **3**, 1–11 (2022).
2. Madhuvilakku, R., Yen, Y. K., Yan, W. M. & Huang, G. W. Laser-scribed Graphene Electrodes Functionalized with Nafion/Fe_3O_4 Nanohybrids for the Ultrasensitive Detection of Neurotoxin Drug Clioquinol. *ACS Omega* (2022) doi:10.1021/acsomega.2c01069.
3. Hsu, H. L. *et al.* Reduction of photoluminescence quenching by deuteration of ytterbium-doped amorphous carbon-based photonic materials. *Materials.* **7**, 5643–5663 (2014).
4. Trai, N. *et al.* Surface features of polymer electrolyte membranes for fuel cell applications: An approach using S_2p XPS analysis. *Sci. Technol. Dev. J.* **24**, 2100–2109 (2021).
5. Li, Y. C. *et al.* Binding Site Diversity Promotes CO_2 Electroreduction to Ethanol. *J. Am. Chem. Soc.* **141**, 8584–8591 (2019).
6. Zhan, C. *et al.* Revealing the CO Coverage-Driven C-C Coupling Mechanism for Electrochemical CO_2 Reduction on Cu_2O Nanocubesvia OperandoRaman Spectroscopy. *ACS Catal.* **11**, 7694–7701 (2021).
7. Monteiro, M. C. O., Dattila, F., López, N. & Koper, M. T. M. The Role of Cation Acidity on the Competition between Hydrogen Evolution and CO_2 Reduction on Gold Electrodes. *J. Am. Chem. Soc.* **144**, 1589–1602 (2022).
8. Cao, Y. *et al.* Surface hydroxide promotes CO_2 electrolysis to ethylene in acidic conditions. *Nat. Commun.* **14**, (2023).
9. Huang, J. E. *et al.* CO_2 electrolysis to multicarbon products in strong acid. *Science (80-.).* **372**, 1074–1078 (2021).
10. Ma, Z. *et al.* CO2 electroreduction to multicarbon products in strongly acidic electrolyte via synergistically modulating the local microenvironment. *Nat. Commun.* **13**, 1–11 (2022).
11. Huang, J. E. *et al.* CO_2 electrolysis to multicarbon products in strong acid. *Science.* **372**, 1074–1078 (2021).
12. Zhao, Y. *et al.* Conversion of CO_2 to multicarbon products in strong acid by controlling the catalyst microenvironment. *Nat. Synth.* **2**, 403–412 (2023).

13. O'Brien, C. P. *et al.* Single Pass CO_2 Conversion Exceeding 85% in the Electrosynthesis of Multicarbon Products via Local CO_2 Regeneration. *ACS Energy Lett.* **6**, 2952–2959 (2021).
14. Gu, J. *et al.* Modulating electric field distribution by alkali cations for CO_2 electroreduction in strongly acidic medium. *Nat. Catal.* **5**, 268–276 (2022).
15. Nie, W., Heim, G. P., Watkins, N. B., Agapie, T. & Peters, J. C. Organic Additive-derived Films on Cu Electrodes Promote Electrochemical CO_2 Reduction to C_{2+} Products Under Strongly Acidic Conditions. *Angew. Chemie Int. Ed.* **62**, e202216102 (2023).
16. Fan, M. *et al.* Cationic-group-functionalized electrocatalysts enable stable acidic CO_2 electrolysis. *Nat. Catal.* **6**, 763–772 (2023).
17. Vichou, E. *et al.* Tuning Selectivity of Acidic Carbon Dioxide Electrolysis via Surface Modification. *Chem. Mater.* **35**, 7060–7068 (2023).
18. Yu, X. *et al.* Coverage enhancement accelerates acidic CO_2 electrolysis at ampere-level current with high energy and carbon efficiencies. *Nat. Commun. 2024 151* **15**, 1–9 (2024).
